# Unraveling *Pseudomonas aeruginosa* and *Candida albicans* Communication in Coinfection Scenarios: Insights Through Network Analysis

**DOI:** 10.3389/fcimb.2020.550505

**Published:** 2020-11-11

**Authors:** Tânia Grainha, Paula Jorge, Diana Alves, Susana Patrícia Lopes, Maria Olívia Pereira

**Affiliations:** CEB—Centre of Biological Engineering, LIBRO—Laboratory of Research in Biofilms Rosário Oliveira, University of Minho, Campus de Gualtar, Braga, Portugal

**Keywords:** *Pseudomonas aeruginosa*, *Candida albicans*, biofilms, polymicrobial, coinfection, interactions, database

## Abstract

Modern medicine is currently facing huge setbacks concerning infection therapeutics as microorganisms are consistently knocking down every antimicrobial wall set before them. The situation becomes more worrying when taking into account that, in both environmental and disease scenarios, microorganisms present themselves as biofilm communities that are often polymicrobial. This comprises a competitive advantage, with interactions between different species altering host responses, antimicrobial effectiveness, microbial pathogenesis and virulence, usually augmenting the severity of the infection and contributing for the recalcitrance towards conventional therapy. *Pseudomonas aeruginosa* and *Candida albicans* are two opportunistic pathogens often co-isolated from infections, mainly from mucosal tissues like the lung. Despite the billions of years of co-existence, this pair of microorganisms is a great example on how little is known about cross-kingdom interactions, particularly within the context of coinfections. Given the described scenario, this study aimed to collect, curate, and analyze all published experimental information on the molecular basis of *P. aeruginosa* and *C. albicans* interactions in biofilms, in order to shed light into key mechanisms that may affect infection prognosis, increasing this area of knowledge. Publications were optimally retrieved from PubMed and Web of Science and classified as to their relevance. Data was then systematically and manually curated, analyzed, and further reconstructed as networks. A total of 641 interactions between the two pathogens were annotated, outputting knowledge on important molecular players affecting key virulence mechanisms, such as hyphal growth, and related genes and proteins, constituting potential therapeutic targets for infections related to these bacterial-fungal consortia. Contrasting interactions were also analyzed, and quorum-sensing inhibition approaches were highlighted. All annotated data was made publicly available at www.ceb.uminho.pt/ISCTD, a database already containing similar data for *P. aeruginosa* and *Staphylococcus aureus* communication. This will allow researchers to cut on time and effort when studying this particular subject, facilitating the understanding of the basis of the inter-species and inter-kingdom interactions and how it can be modulated to help design alternative and more effective tailored therapies. Finally, data deposition will serve as base for future dataset integration, whose analysis will hopefully give insights into communications in more complex and varied biofilm communities.

## Introduction

In real scenarios, including natural, clinical, and industrial settings, microorganisms team up to survive the hostile environmental conditions they are continuously exposed to by building complex communities called biofilms. Their organization in these surface-attached microbial communities, embedded in a self-produced matrix, facilitates the intercellular exchange of metabolites, genetic material, and signaling molecules (Flemming et al., 2016). Once established, biofilms are less susceptible to predatory action and antimicrobial agents compared to their planktonic counterparts (Flemming and Wingender, 2010). In addition to this advantageous form of microbial life, one type of microorganisms often exists in close association with other microbial species. Interactions between different microorganisms may provide an advantage to one party (antagonism) or both parties (mutualism) (Dhamgaye et al., 2016). Although most natural biofilms are polymicrobial, most of our current knowledge about these communities comes from the study of single-species biofilms, using model bacteria (Coenye and Nelis, 2010; Lebeaux et al., 2013). Recognition of the real impact of polymicrobial biofilms has triggered a recent interest towards the study of multi-species communities to learn more about their behavior (Short et al., 2014).

This is particularly true for the clinical field, since the human body is well known to be colonized by trillions of microorganisms pertaining to more than 10,000 different species (Peleg et al., 2010; Potera, 2014; Bai et al., 2019). Examples of human polymicrobial diseases include cystic fibrosis (CF) lung infection, otitis media, biomaterial-associated infections, urinary tract infections, periodontitis, wound infections, and diabetic ulcers (Short et al., 2014). The interactions between the microorganisms comprising polymicrobial communities can exacerbate the severity of infections, compromising their antimicrobial treatments (Peters et al., 2012; De Vos et al., 2017). Among the great panoply of interactions found within the context of human infections, communication between fungi and bacteria has been the focus of great interest in the last years (Shirtliff et al., 2009; Diaz et al., 2014). *Candida albicans* and *Pseudomonas aeruginosa* comprise an example of a clinically relevant fungal-bacterial consortium commonly found in the respiratory tract and skin (Dhamgaye et al., 2016).

### Pathogenesis of *P. aeruginosa* and *C. albicans*

*P. aeruginosa* is a motile Gram-negative bacterium ubiquitously found in nature, including aquatic, terrestrial, animal, plant, and human environments. It is an opportunistic pathogen with the ability to cause life-threatening acute and chronic infections, especially in hospitalized and immunocompromised individuals, such as CF lung infection, ventilator associated pneumonia (VAP), burn wounds, keratitis, otitis media, and urinary tract and gastrointestinal infections (Moradali et al., 2017; Azam and Khan, 2019). There are essentially three key factors for *P. aeruginosa* pathogenic abilities: production of virulence factors (VF), resistance to antimicrobial agents, and biofilm formation (Gellatly and Hancock, 2013).

*P. aeruginosa* is widely recognized for producing an arsenal of VF associated to diverse functions, such as motility (e.g. flagella, pilli), tissue invasion, and damage to the host cells [e.g. proteases such as elastase, alkaline phosphatase, hemolysins, pyocyanin, siderophores, endotoxin A, lipopolysaccharide (LPS), exotoxin A], as well as surface adhesion and biofilm formation (e.g. alginate, Pel, Psl, lectins) (Hauser, 2011; Strateva and Mitov, 2011; Moradali et al., 2017). In addition to these factors, *P. aeruginosa* is known for its remarkable ability to resist antibiotics. Actually, the World Health Organization (WHO) has listed carbapenem-resistant *P. aeruginosa* in the top three species for which the development of new treatments is in critical need (WHO, 2017). The major resistance mechanisms of *P. aeruginosa* that allow it to withstand the action of antibiotics can be intrinsic or innate (e.g. restricted outer membrane permeability, efflux pumps, antibiotic-degrading enzymes), acquired by either horizontal transfer of resistance genes or by mutations, or adaptive (e.g. biofilm formation, persister cells) (Pang et al., 2019). The ability of *P. aeruginosa* to form biofilms comprises an advantage in many infections (Mulcahy et al., 2014). In effect, it is considered the hallmark of chronic infections and revealing of disease progression. The biofilm environment triggers the development of small colony variants and persister cells that can resist higher doses of antibiotics (Burrows, 2018; Pestrak et al., 2018).

*C. albicans* is a polymorphic fungus that has the ability to grow in several different morphological forms, namely yeast, hyphae, and pseudohyphae, depending on the environmental conditions (Han et al., 2011). This microorganism is frequently found as part of the normal microbiota of the skin, gastrointestinal tract, and female genital tract, being harmless in the majority of cases (Morales and Hogan, 2010). However, *C. albicans* can take advantage of immunocompromised patients and cause several opportunistic infections (Harriott and Noverr, 2011). Similarly to *P. aeruginosa*, there are three important features for the pathogenesis of *C. albicans*: its ability to switch from yeast-to-filamentous growth and vice versa, secretion of VF, and biofilm formation (Poulain, 2015).

Each morphology assumed by *C. albicans* provides different advantages in the development of infection. The filamentous forms (hyphae and pseudohyphae) are one of its most important virulence mechanisms (VM), being responsible for host cell invasion and macrophage destruction, thus having an important role on the establishment of an infection process (Vylkova and Lorenz, 2017; Desai, 2018). In turn, the yeast form is believed to be important for dissemination through the bloodstream and adhesion to endothelial surfaces (Seman et al., 2018). This phenotypic switch can be induced by temperature, pH, nutrient concentration, cell density, and human serum (Sudbery, 2011). Besides this morphological plasticity, there are other VF contributing for *C. albicans* pathogenesis, such as adhesins (biomolecules that enable binding to the host cells) and hydrolytic enzymes (e.g. aspartyl proteinases and phospholipases) (Calderone and Fonzi, 2001; Dadar et al., 2018). It is now well acknowledged that biofilm formation is one of the main virulence features involved in the pathogenesis of *C. albicans*, as these organized communities confer protection against antimicrobial therapy and host defenses (Wall et al., 2019).

### Quorum Sensing in *P. aeruginosa* and *C. albicans*

Microorganisms interact by means of a major process that allows them to coordinately sense and respond to the fluctuating conditions of the surrounding environment. This cell-to-cell communication is called quorum-sensing (QS) and small diffusible signaling molecules, termed autoinducers (AI), mediate it. AI regulate the expression of target genes, namely those related to virulence, pathogenicity, resistance, and competition, when a threshold concentration is reached in accordance with population density (Fuqua et al., 1994; Dixon and Hall, 2015; Hawver et al., 2016; Paul et al., 2018). AI-mediated signaling in QS allows microbial communication and the control of pivotal processes, namely VF production, biofilm formation, motility, sporulation, production of secondary metabolites, and stress adaptation through, for example, secretion systems (Pena et al., 2019).

The structure and functioning of the QS machineries deployed by *P. aeruginosa* have been well elucidated (Jimenez et al., 2012; Lee and Zhang, 2015; Magalhães et al., 2019). *P. aeruginosa* possesses four well-known QS systems: LasI/LasR, RhlI/RhlR, PqsABCDE/PqsR, and AmbBCDE/IqsR. Each of these systems produces an AI, namely 3-oxododecanoyl-L-homoserine lactone (3-oxo-C12-HSL), N-butanoyl homoserine lactone (C4-HSL), 2-heptyl-3-hydroxy-4-quinolone (Pseudomonas Quinolone Signal—PQS), and 2-(2-hydroxyphenyl)-thiazole-4-carbaldehyde (Integrated Quorum Sensing Signal—IQS), respectively (Magalhães et al., 2019). The hierarchical regulation of *P. aeruginosa* QS systems allows triggering massive changes in genetic expression, namely in genes involved in motility, biofilm formation, iron sequestration, antibiotic resistance, and immune system evasion (Shrout et al., 2006; Jakobsen et al., 2013). For example, the LasI/LasR system controls the production of multiple VF involved in acute infection and host cell damage, such as LasA and LasB elastases, exotoxin A, and alkaline protease (Gambello et al., 1993; Jones et al., 1993; Passador et al., 1993). It also controls the expression of a major biofilm matrix component, Pel (Ueda and Wood, 2009). In turn, the excretion of bacterial biosurfactants, such as rhamnolipids, is regulated by the Rhl system (Pearson et al., 1995), while the PqsABCDE/PqsR system controls biofilm formation and its structural stability through extracellular DNA and lectin production (Allesen-Holm et al., 2006; Lee and Zhang, 2015).

Contrary to the enormous information available about QS in prokaryotes, QS in the fungal kingdom was somewhat concealed until the discovery of farnesol, a common sesquiterpene produced by *C. albicans* and similar in structure to bacterial 3-oxo-C12-HSL, in 2001 (Hornby et al., 2001). Although other molecules mediating QS in *C. albicans* have been identified since then, including farnesoic acid (Oh et al., 2001) and aromatic amino acid derived alcohols like tyrosol (Chen et al., 2004), tryptophol, and phenylethanol (Lingappa et al., 1969; Chen and Fink, 2006), farnesol is undoubtedly the most explored AI. Regulation of fungal virulence by farnesol is thought to be particularly confined to inhibit yeast-to-hypha transition (Dižová and Bujdáková, 2017), to promote reverse morphogenesis (Polke and Jacobsen, 2017), and to inhibit biofilm development (Khan et al., 2020). Farnesol is known to directly inhibit the fungal Ras1-cyr1-cAMP-protein kinase A (PKA) signaling pathway, ultimately blocking cAMP synthesis and consequently suppressing hyphal development (Davis-Hanna et al., 2007; Hall et al., 2011; Grahl et al., 2015). Following the same event cascade, the catalytic subunits of PKA, Tpk1, and Tpk2, are also stimulated. Tpk1 and Tpk2 share redundant functions in hyphal growth, adhesion, and biofilm formation, but also have distinct roles in stress responses and pathogenesis, respectively (Lindsay et al., 2012; Lin et al., 2018). *RAS1, CYR1*, and *EFG1* (other important hypha-associated genes) have also been documented as important effector genes targeted by farnesol (Grainha et al., 2018).

Apart from farnesol, the AI farnesoic acid, tyrosol, tryptophol, and phenylethanol may also affect important processes such as morphogenesis, biofilm development, limitation of cell population density, control of nutrient competition, and control of infection dissemination (Wongsuk et al., 2016). Tyrosol, for instance, stimulates *C. albicans* filamentation, biofilm formation, and germ tube development (Chen et al., 2004; Alem et al., 2006), in contrast to farnesol. Sadly, understanding their underlying regulatory mechanisms is yet to be fully elucidated.

### Interactions Between *P. aeruginosa* and *C. albicans*

Bacteria and fungi often coexist in competitive ecological niches in a myriad of ways, communicating through excretion of metabolic by-products, physical interactions, chemical signaling exchanges, and alterations in the environment (Peleg et al., 2010). *P. aeruginosa* and *C. albicans* are likely the best-studied models in the investigation of these inter-kingdom interactions in clinical context, with reports on their impressive interactions and communication having greatly evolved in the last decade (Ader et al., 2011; Roux et al., 2013; Trejo-Hernández et al., 2014; Kumar et al., 2017; Rodrigues et al., 2017). Although *P. aeruginosa-C. albicans* interplay is fundamentally antagonistic, its interaction is rather complex, as synergistic and antagonistic effects can occur simultaneously, mostly dictated by physical associations and by secreted factors *via* QS (Fourie et al., 2016; Fourie and Pohl, 2019; Nogueira et al., 2019).

As stated, *P. aeruginosa* and *C. albicans* are frequently co-isolated in polymicrobial infections, such as those related to the skin, the lungs, and to medical devices (Méar et al., 2013). Their genetic and phenotypic plasticity, along with their propensity to assemble as recalcitrant polymicrobial biofilms, places a considerable burden in infections in which they are involved, explaining the increasing interest and the bulk of research on this topic. For example, it has been well documented that *P. aeruginosa* and *C. albicans* colonize the lungs of CF patients and readily form biofilms on endotracheal tube surfaces (Harriott and Noverr, 2011; Fourie et al., 2016). Interestingly, *C. albicans* is only implicated in airway colonization in critically ill (elderly, immunocompromised, and/or hospitalized) individuals undergoing invasive mechanical ventilation. However, and despite their antagonistic relationship, those who display *C. albicans* tracheobronchial colonization are at increased risk of acquiring severe VAP infections due to *P. aeruginosa* (Hamet et al., 2012).

#### Effect of *P. aeruginosa* on *C. albicans*

**Figure 1:**
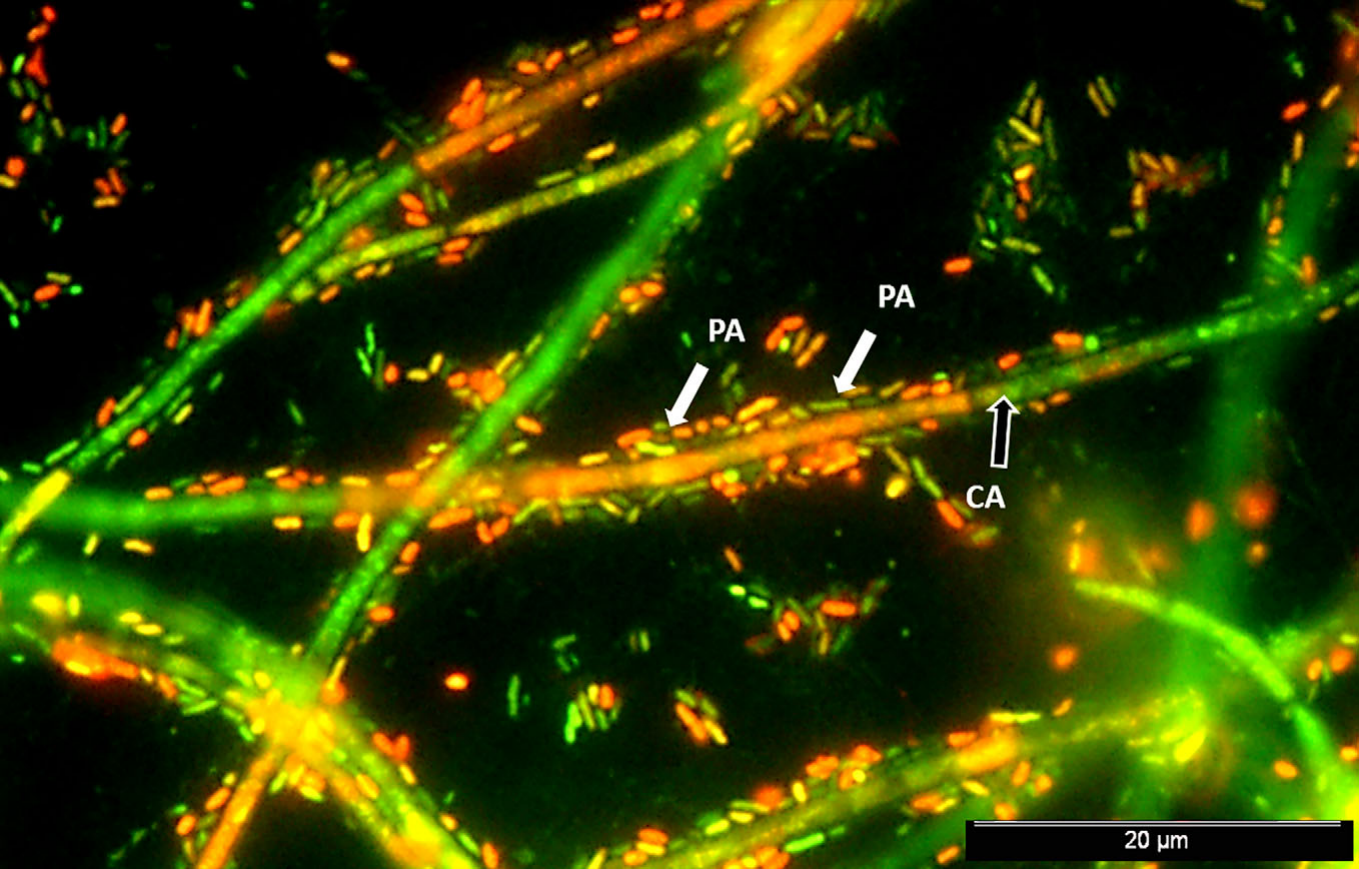
Mixed *P. aeruginosa-C. albicans* 24 h–old biofilm. Epifluorescence image of a stained biofilm with a mixture of SYTO® BC and propidium iodide (PI) (Invitrogen™, CA, USA) shows *P. aeruginosa* PAO1 (PA) cells (highlighted by white arrows) surrounding and colonizing in a great extension the *C. albicans* SC5314 (CA) hyphae (black arrow). Cells were prepared at approximately 10^7^ CFU/mL in RPMI 1640 media and mixed in equal proportions. Mixed suspensions were transferred to Thermanox® plastic coverslips, placed in 24-well plates, and incubated at 120 rpm and 37°C for 24 h prior to biofilm analysis.

The inhibitory effect of *P. aeruginosa* on *C. albicans* growth was first reported in the 1970s (Hughes and Kim, 1973; Auger and Joly, 1977). In 2002, Hogan and Kolter reported killing of *C. albicans* hyphal cells by *P. aeruginosa*, showing, however, no such effect on fungal yeasts (Hogan and Kolter, 2002). The deadly effect imparted by *P. aeruginosa* was further confirmed to be largely dependent on the distinct morphotypes exhibited by *C. albicans* (Kerr et al., 1999; Hogan and Kolter, 2002; Hogan et al., 2004). Often, a reversion of germ tube formation may occur in the presence of *P. aeruginosa*. The most common event elucidating the physical interaction among *P. aeruginosa* and *C. albicans* is likely the extensive bacterial attachment to fungal hyphae (Ovchinnikova et al., 2012; Fourie et al., 2016). **Figure 1** displays a mixed *P. aeruginosa-C. albicans* 24 h–old biofilm wherein *C. albicans* filamentous populations served as a scaffold for *P. aeruginosa* attachment (data not published). Physical association among *P. aeruginosa* and *C. albicans* occurs via cell wall-associated compounds in bacteria, such as type IV pili, lectin-carbohydrate interactions, and mannans (Brand et al., 2008). The bacterial attachment to fungal hyphae is hypothesized to be caused by nutrient competition at the first hour of co-isolation, after which the bacterial-fungal interaction tends to be of parasitism (Trejo-Hernández et al., 2014). Secreted LPS, another bacterial cell-wall component, also functions on polymicrobial intertwining, by interfering with fungal filamentation, metabolism, and growth (Bandara et al., 2010; Bandara et al., 2013).

The *P. aeruginosa* QS signal 3-oxo-C12-HSL plays major roles in interaction regarding the bacterial binding to *C. albicans* filaments and in the inhibition of yeast to hyphae switch (Hogan et al., 2004; Ovchinnikova et al., 2012). Because of the inhibition of yeast-to-hypha transformation, *C. albicans* gains compromised ability to adhere or invade tissues (Maza et al., 2017). The 2-heptyl-4-quinolone (HHQ), the immediate precursor of the PQS signal, has been shown to repress *C. albicans* biofilm formation (Reen et al., 2011). PQS induces the expression of the VF phenazines, such as pyocyanin, which are toxic products that produce a deleterious effect in eukaryotic cells (Kaleli et al., 2007; Phelan et al., 2014). This effect may be associated with the generation of highly toxic reactive oxygen species (ROS) (O’Malley et al., 2003; Gloyne et al., 2011). Pyocyanin was shown to reduce cAMP (Kerr et al., 1999), which is required for yeast-to-hyphae transition (Gibson et al., 2009; Tupe et al., 2015). The effect of phenazines, including pyocyanin, in disturbing *C. albicans* biofilm formation and hyphal growth has also been evidenced (Morales et al., 2013; Lindsay et al., 2014). Certain phenazines have even showed potential to act synergistically in concert with several azole agents against fungal infection (Kumar et al., 2014). Along with secreting inhibitory molecules, *P. aeruginosa* also produces substances such as the proteolytic enzyme elastase LasB, which increases the virulence of *C. albicans* (Peleg et al., 2010).

#### Effect of *C. albicans* on *P. aeruginosa*

*C. albicans* secreted factors, such as farnesol, tyrosol, ethanol, oxylipins, and eicosanoids, have been shown to affect *P. aeruginosa* growth and biofilm. *C. albicans* generally uses farnesol to resist oxidative stress and to induce the generation of ROS, thus providing competitive advantage over bacteria (Westwater et al., 2005). Farnesol has a deleterious effect on *P. aeruginosa*, modulating PQS-controlled virulence in a dose-dependent manner (Cugini et al., 2007). Farnesol is able to inhibit pyocyanin (Cugini et al., 2007) and rhamnolipid-mediated swarming motility (McAlester et al., 2008) through such modulation, while also inhibiting other virulence-related proteins (Abdel-Rhman et al., 2015).

Tyrosol, another *C. albicans* AI, has been shown to inhibit secretion of haemolysin and protease (toxins usually involved in tissue damage) by *P. aeruginosa* at high concentrations (Abdel-Rhman et al., 2015). Ethanol is a common fermentation product produced by many bacteria and fungi known to influence *P. aeruginosa* in diverse polymicrobial settings. In the context of infections where *P. aeruginosa* coexists with *C. albicans*, exogenous fungal-produced ethanol may alter phenazine production and promote biofilm development on biotic and abiotic surfaces. In addition, ethanol enhances bacterial Pel matrix production and represses surface motility (Lewis et al., 2019). Its production is continuously stimulated by the enhanced *P. aeruginosa* biofilm formation and production of antifungal phenazines, in a positive feedback loop (Chen et al., 2014). Eicosanoids (e.g. prostaglandin E2, PGE2) are often secreted by *Candida* spp., including *C. albicans*. Although the role of such fatty acid metabolites is still to be determined in the bacterial-fungal cross talk, it has been suggested that they act as immunomodulatory mediators that affect the dynamics of mixed bacterial-fungal infections and their outcome (Fourie et al., 2016; Fourie et al., 2017). Competition for iron is also one of the main antagonistic interactions occurring in *P. aeruginosa-C. albicans* infections. Enhanced secretion of *P. aeruginosa* siderophore pyoverdine in mixed species biofilms leads to a decrease in iron availability on *C. albicans* and consequently to inhibition of yeast growth (Purschke et al., 2012).

Given the abovementioned information, the purpose of this study was to gather, curate, and analyze all published experimental data regarding the molecular basis of *P. aeruginosa* and *C. albicans* interactions, with emphasis on biofilms. The objective is to provide insights into the key communication mechanisms and molecular players affecting *P. aeruginosa* and *C. albicans* coexistence, hopefully facilitating the current understanding of inter-species and inter-kingdom interplays, and ultimately guide the design for novel and effective tailored therapies.

## Materials and Methods

### Information Retrieval

Medline (PubMed) database (NCBI, 2004) and Web of Science (Clarivate, 2020) were searched in order to extract information from the scientific literature related to *P. aeruginosa* and *C. albicans* interactions. The scope of the search was optimized so that relevant papers were not overlooked but not so broad that irrelevant papers made the curation effort infeasible. Documents were narrowed down to those whose title, abstract or MeSH (Medical Subject Headings) terms mentioned “*Pseudomonas aeruginosa*”, “*Candida albicans*” and, at least, one term related to interaction (e.g. “co-cultivation”, “mixed biofilms”, “double species biofilms”, “multispecies”, “polymicrobial”, “consortia”, “communication”, “quorum-sensing”).

### Selection of Studies and Data Extraction

Articles searched on PubMed and Web of Science were classified as relevant or irrelevant by analyzing each title and abstract for its suitability for the subject of *P. aeruginosa*-*C. albicans* interactions. When necessary, a full-text article analysis was performed to confirm its relevance. Irrelevant articles were deemed as those out of scope, but also reviews, as they would output duplicated data, and non-English written articles, as they would not be possible to annotate. Out of scope articles were those that did not include testing of the two species in combination or of the effect of exoproducts of one species on the other. Many articles were deemed irrelevant as specific terms pertaining to polymicrobial biofilms included in the query (see *Information Retrieval*) were only mentioned as part of the introduction or contextualization.

As for relevant publications, the full-texts were curated and important information was annotated, namely the interactions between the two microorganisms and, where possible, the molecular entities behind such phenomenon. Accessory information was also annotated, such as strains, mode of growth, and experimental methods. The entities responsible for the annotated interactions and the interaction outcomes were classified into categories, as performed previously (Magalhães et al., 2019). Briefly, interactions were classified into up- or downregulation (for gene and protein targets), stimulation or inhibition (for VM, VF, AI, and other targets), and indifference (for all target entity categories).

### Network Reconstruction and Data Deposition

Data on *P. aeruginosa*-*C. albican*s interactions was reconstructed as networks using Cytoscape (Shannon et al., 2003). Different node colors and shapes were used to depict distinct source organisms and entity categories, respectively. All annotated data was made publicly available in the Inter Species CrossTalk Database (ISCTD) (www.ceb.uminho.pt/ISCTD) (Magalhães et al., 2019), which was previously built to deposit data on communication between microorganisms and was first loaded with information on the interactions between *P. aeruginosa* and *Staphylococcus aureus* obtained in a similar fashion as the present work.

## Results

### Exploring the ISCTD

**Figure 2:**
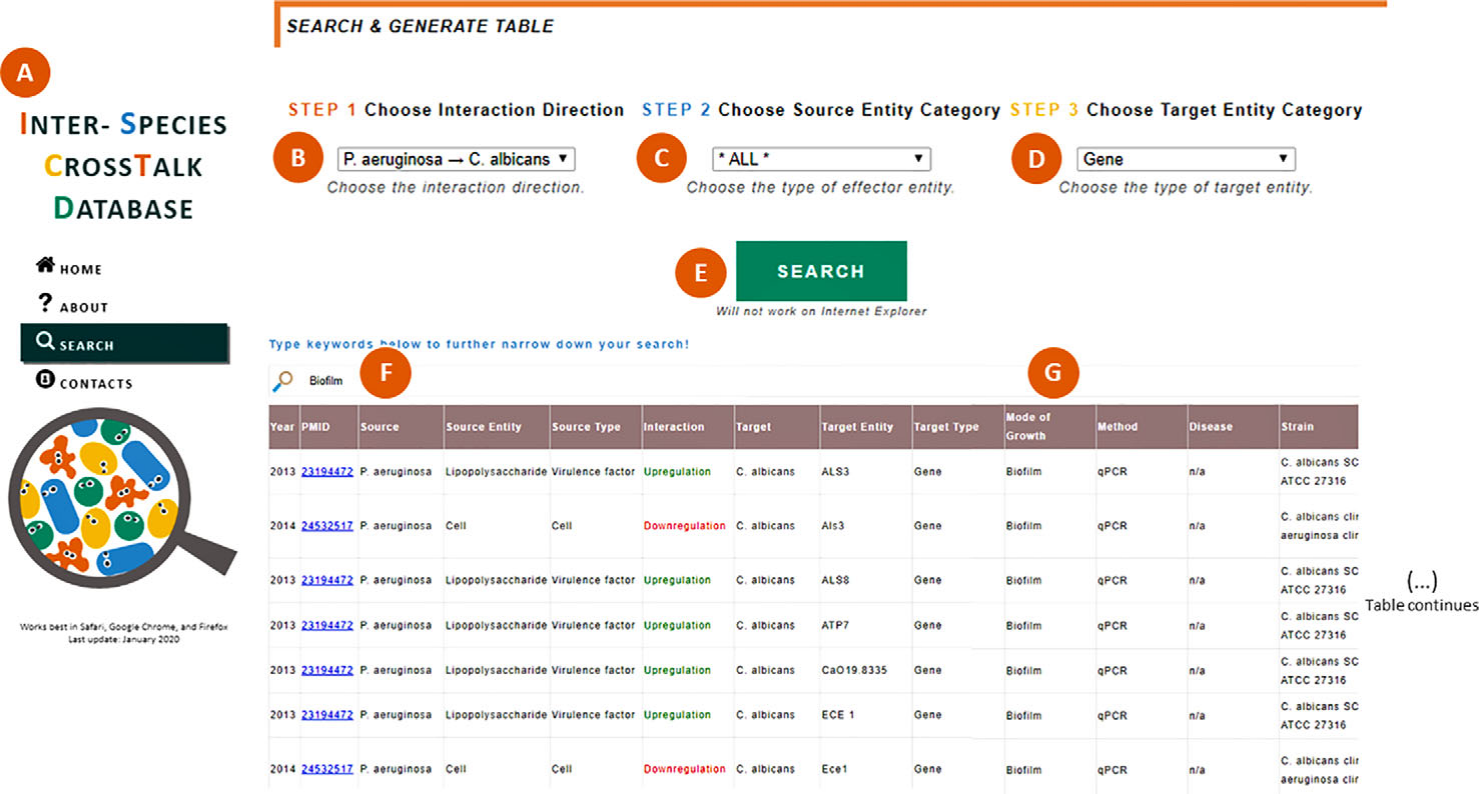
Searching within the ISCTD. **(A)** Navigation panel that allows users to jump within the webpage, including to the “Search” section. **(B)** Selection of the interaction direction of interest, here exemplified by the effect of *P. aeruginosa* on *C. albicans*. **(C)** Selection of the source entity category, which can be left unspecified by selecting the option “All”. **(D)** Selection of the target entity category, here exemplified by “gene”. **(E)** When clicking the “Search” button, a table is generated containing all the information annotated by the expert curators and sifted through the user’s selection in **(B–D)**. **(F)** Users can type keywords to further specify their search, here exemplified by “biofilm”. **(G)** Table is narrowed down to show only interactions observed in biofilms.

The ISCTD (www.ceb.uminho.pt/ISCTD) (Magalhães et al., 2019) now provides public access to the annotated information about *P. aeruginosa* and *C. albicans* interactions. Users can specify search preferences by selecting the direction of the interaction (*P. aeruginosa* > *C. albicans* or *C. albicans* > *P. aeruginosa*) and, optionally, the source and target entity categories. The respective data selection is shown in a table as an organized view of all interaction details, such as strains, modes of growth, experimental methods, and observations made by the expert curators. Links to PubMed records are always included so users can easily access the original publications. Users can further explore and narrow down the selected data by searching specific terms within the table, such as a specific mode of growth (e.g. “biofilm”), a VF (e.g. “pyoverdine”), or a gene (e.g. “*lasA*”). An example of a search focused on the effect of *P. aeruginosa* on *C. albicans*’ genes in biofilms is illustrated in **Figure 2**.

### Network Overview

The elaboration of the *P. aeruginosa*-*C. albicans* network comprised the analysis of 164 PubMed and 215 Web of Science documents (dating from June 1975 to July 2020), of which 60 were exclusive to the first and 108 to the latter. A total of 29 documents (dating from December 2004 until May 2020) (complete reference list in **Supplementary Material**) were considered relevant. The annotation of these relevant studies outputted a total of 641 interactions between the two pathogens, in which 54% were related to the effect of *P. aeruginosa* on *C. albicans* and 46% to the opposite effect.

**Figure 3:**
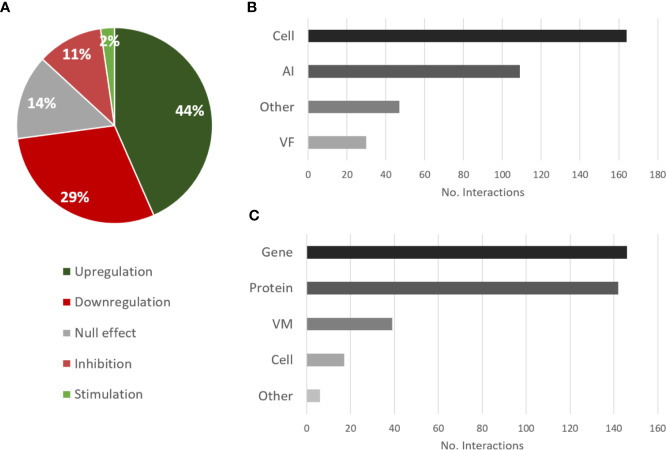
Overview of the types of interactions and entities annotated for the effects of *P. aeruginosa* on *C. albicans*. **(A)** Proportional data on the types of interactions; **(B)** Total number of interactions annotated for each source category; **(C)** Total number of interactions annotated for each target category. AI, autoinducer; VM, virulence mechanism; VF, virulence factor.

The most annotated type of interaction illustrating the effect of *P. aeruginosa* on *C. albicans* was upregulation followed by downregulation (**Figure 3A**), which reflects the great number of proteins and genes that are usually analyzed in studies where methods such as Matrix-Assisted Laser Desorption/Ionization-Time Of Flight (MALDI-TOF) and RNA sequencing are used. These high-throughput studies also explain the great number of interactions annotated for proteins and genes as targets (**Figure 3C**). However, this does not translate to a majority of papers using these techniques. In fact, only 45% of annotated papers studying the effect of *P. aeruginosa* on *C. albicans* analyzed protein or gene expression, and only 41% used these types of high-throughput methods. Regarding the source type, the effect of most interactions was reported for “cell” as the source entity (**Figure 3B**), which means that the molecular effector entity of the interaction was not studied in detail.

**Figure 4:**
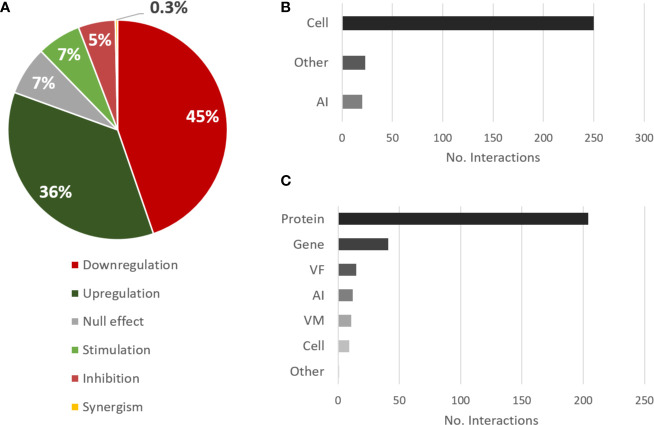
Overview of the types of interactions and entities annotated for the effects of *C. albicans* on *P. aeruginosa*. **(A)** Proportional data on the types of interactions; **(B)** Total number of interactions annotated for each source category; **(C)** Total number of interactions annotated for each target category. Legend: AI, autoinducer; VM, virulence mechanism; VF, virulence factor.

As for the effect of *C. albicans* on *P. aeruginosa*, the trend is very similar to the opposite direction. The most annotated interactions were downregulation and upregulation (**Figure 4A**), although a preponderance of downregulation is unveiled here, while the most annotated source and target types were cell and protein, respectively (**Figures 4B, C**), by the same reasons previously mentioned.

### Effect of QS Molecular Players on *P. aeruginosa*-*C. albicans* Interactions

Four different AI from *P. aeruginosa* were annotated for their effect on *C. albicans*. For instance, 3-oxo-C12-HSL was annotated as inhibiting the hyphal form of the fungus (Hogan et al., 2004; Hall et al., 2011), the secondary messenger cAMP (Hall et al., 2011), the cAMP synthesis-related enzyme Cyr1 (Hogan et al., 2004), and biofilm formation, while stimulating *C. albicans* adhesion to mammalian cells (Curutiu et al., 2017) (**Table 1**). As previously mentioned, the morphogenesis of *C. albicans* is largely regulated through the cAMP/PKA pathway, meaning that 3-oxo-C12-HSL is able to inhibit yeast to hyphae transition by affecting it. Additionally, 3-oxo-C12-HSL was also shown to downregulate the expression of *ECE1*, *HWP1*, and *SAP5*, all hyphae-specific genes (**Table 1**) (Hogan et al., 2004). As stated, hyphae development by *C. albicans* is one of its most important VM, having an important role on the establishment of the infection process, which is hindered by 3-oxo-C12-HSL. Although this effect may be apparently detrimental for the infection, the reversion or maintenance of the fungus in the yeast form may enhance its dissemination capabilities and spread the infection to other areas of the host. However, it has also been shown that the presence of 3-oxo-C12-HSL, at sub-growth and sub-hyphal inhibitory concentrations, favorably affects *C. albicans* when challenged with fluconazole by upregulation of genes known to be associated with antimicrobial resistance (e.g. *GAL102*, *MDR2*, *INO2*, *ADA2*) (Bandara et al., 2020) (**Table 1**).

**Table 1:** Effect of AI on *P. aeruginosa*-*C. albicans* interactions.

Source	AI	Interaction	Target	Ref.
*P. aeruginosa*	3-oxo-C12-HSL	Inhibition	Hyphae^(p)^, cAMP^(p)^, Cyr1^(p)^, Biofilm^(b)^	(Hogan et al., 2004; Hall et al., 2011; Curutiu et al., 2017)
		Stimulation	Adhesion^(b)^	(Curutiu et al., 2017)
		Downregulation	*ECE1*^(p)^, *HWP1*^(p)^, *SAP5*^(p)^, *CDR1*^(b)^*, MDR1*^(b)^*, C6_02100W_A*^(b)^*, CRH11*^(b)^*, FBA1*^(b)^*, IFR2*^(b)^, *MNN12*^(b)^*, PHHB*^(b)^*, SOD5*^(b)^*, TEL1*^(b)^	(Hogan et al., 2004; Hall et al., 2011; Bandara et al., 2020)
		Upregulation	*CDR2*^(b)^, *AAF1*^(b)^, *ADA2*^(b)^, *ADH3*^(b)^, *ALK2*^(b)^, *ALS7*^(b)^, *ARD*^(b)^, *ATX1*^(b)^, *AXL1*^(b)^, *BCR1*^(b)^, *C1_01130W_A*^(b)^, *C1_01510W_A*^(b)^, *C1_03990W_A*^(b)^, *C1_04010C_A*^(b)^, *C1_09210C_A*^(b)^, *C2_01750C_A*^(b)^, *C2_02920W_A*^(b)^, *C2_03690C_A*^(b)^, *C2_09880C_A*^(b)^, *C3_00360W_A*^(b)^, *C3_02630C_A*^(b)^, *C3_03460C_A*^(b)^, *C3_04330C_A*^(b)^, *C3_05450C_A*^(b)^, *C4_02740W_A*^(b)^, *C4_03020W_A*^(b)^, *C5_04030W_A*^(b)^, *C6_00110C_A*^(b)^, *C6_00290W_A*^(b)^, *C6_00920W_A*^(b)^, *C7_00770W_A*^(b)^, *C7_04090C_A*^(b)^, *CDR4*^(b)^, *CR_00040C_A*^(b)^, *CR_05860W_A*^(b)^, *CR_06140W_A*^(b)^, *CR_06960W_A*^(b)^, *CR_07480W_A*^(b)^, *CR_09100C_A*^(b)^, *CR_10230W_A*^(b)^, *CRZ2*^(b)^, *CSH1*^(b)^, *CUP9*^(b)^, *EFG1*^(b)^, *ERO1*^(b)^, *GAL102*^(b)^, *GOR1*^(b)^, *GRP2*^(b)^, *HAL9*^(b)^, *HSP104*^(b)^, *HSP78*^(b)^, *HSP90*^(b)^, *IFD6*^(b)^, *INO2*^(b)^, *ISA1*^(b)^, *LPG20*^(b)^, *MHP1*^(b)^, *MOH1*^(b)^, *NRG1*^(b)^, *OPT3*^(b)^, *PGA52*^(b)^, *RFG1*^(b)^, *RGS2*^(b)^, *RME1*^(b)^, *RPN4*^(b)^, *SIS1*^(b)^, *SNQ2*^(b)^, *SRR1*^(b)^, *STI1*^(b)^, *UGT51C1*^(b)^, *WOR4*^(b)^, *YIM1*^(b)^, *YOR1*^(b)^, *ZCF1*^(b)^, *ZCF39*^(b)^	(Bandara et al., 2020)
	C4-HSL	Inhibition	Biofilm^(b)^	(Curutiu et al., 2017)
		Stimulation	Adhesion^(b)^	
	HHQ	Inhibition	Biofilm^(b)^	(Reen et al., 2011; Curutiu et al., 2017)
		Stimulation	Adhesion^(b)^	(Curutiu et al., 2017)
		Null effect	Hyphae^(p)^, Adhesion^(b)^	(Reen et al., 2011)
	PQS	Inhibition	Biofilm^(b)^	(Curutiu et al., 2017)
		Stimulation	Adhesion^(b)^	
		Null effect	Hyphae^(p)^	(Reen et al., 2011; Watrous et al., 2013)
*C. albicans*	Farnesol	Inhibition	PQS^(p)^, Adhesion^(b)^, Swarming motility^(p)^, Pyocyanin^(p)^, Haemolysin^(p)^, Cell^(p)^	(Cugini et al., 2007; McAlester et al., 2008; Abdel-Rhman et al., 2015)
		Downregulation	*pqsA*^(p)^, *pqsR*^(p)^	(Cugini et al., 2007)
		Null effect	Cell^(p);(v)^, *pqsR*^(p)^, Proteases^(p)^	(Cugini et al., 2007; Abdel-Rhman et al., 2015; Lopez-Medina et al., 2015)
		Upregulation	*pqsH*^(b)^	(Cugini et al., 2010)
		Stimulation	HHQ^(b)^, PQS^(b)^, C4-HSL^(b)^, Pyocyanin^(b)^	
	Tyrosol	Inhibition	Haemolysin^(p)^, Proteases^(p)^, Cell^(p)^	(Abdel-Rhman et al., 2015)

p, planktonic; b, biofilm; v, in vivo.

Concerning other AI, C4-HSL, PQS, and its precursor HHQ were also annotated as interfering with biofilm formation on inert surfaces but to stimulate *C. albicans* adhesion to a cellular substratum (Curutiu et al., 2017) (**Table 1**). This switch from antagonistic to synergistic interactions is dependent on the host, which highlights the need for better understanding the role played by the latter in polymicrobial infections. PQS and HHQ have multifunctional roles in QS and iron uptake, playing a key role in coordinating virulence in *P. aeruginosa* (Diggle et al., 2007). Although the annotated effect of these molecules on *C. albicans* hyphae was null (Reen et al., 2011; Watrous et al., 2013), HHQthey seems to interfere with biofilm formation (**Table 1**) (Reen et al., 2011; Curutiu et al., 2017). Overall, there is a clear negative effect on the phenotypic switching and biofilm formation of *C. albicans* mediated through QS of *P. aeruginosa* when the host is not considered.

Regarding the two annotated AI of *C. albicans*, farnesol, and tyrosol, the former seems to affect the production of AI from *P. aeruginosa*, namely PQS (Cugini et al., 2007; Cugini et al., 2010), HHQ, and C4-HSL (Cugini et al., 2010) (**Table 1**). Farnesol is also involved in the inhibition of different VM and VF of this bacterium, such as adhesion, swarming motility, and haemolysin production (Cugini et al., 2007; McAlester et al., 2008; Abdel-Rhman et al., 2015) (**Table 1**). Distinct farnesol effects were also observed between planktonic and biofilm growth for pyocyanin production (**Table 1**). Tyrosol was also annotated as affecting the production of VF of *P. aeruginosa*, namely the inhibition of the production of haemolysin and proteases (Abdel-Rhman et al., 2015) (**Table 1**). These two exoenzymes greatly influence the pathogenicity of *P. aeruginosa*, contributing to infection establishment through elastin degradation and vascular permeability, respectively (Azam and Khan, 2019).

Overall, it is safe to say that QS in both pathogens is a key factor mediating their antagonistic relationship, with their capacity for infection establishment being one of their most affected traits.

### Interaction Effects on Virulence Mechanisms and Virulence Factors

**Figure 5:**
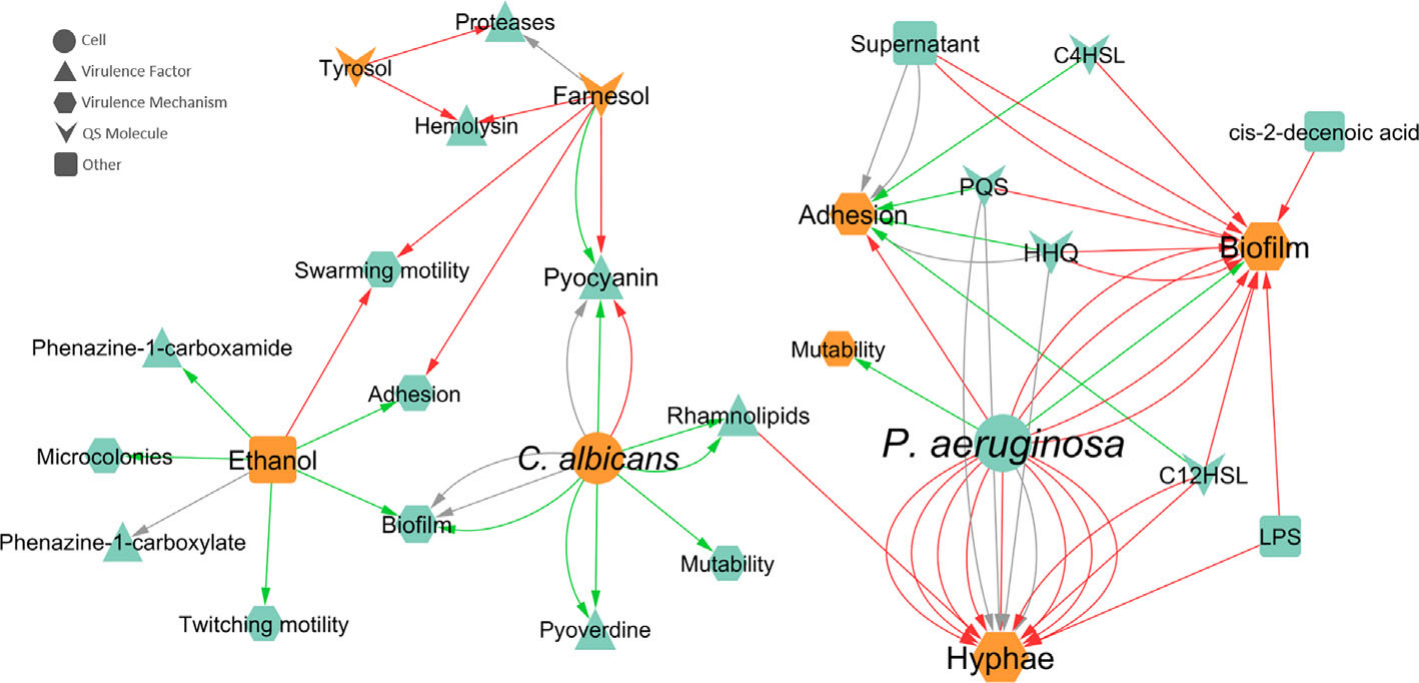
Network of the effects of *P. aeruginosa*-*C. albicans* interactions on VF and VM. Teal nodes, *P. aeruginosa*; orange nodes, *C. albicans*; green arrows, stimulation; grey arrows, null effect; red arrows: inhibition; node and node label sizes are directly proportional to the number of related (outward and inward) edges (interactions).

The influence of inter-species interactions on the expression of VM and VF can greatly affect the severity of the polymicrobial infection. Given their importance, two networks were constructed regarding the effects of one species on the virulence of the other. Concerning the effect of *P. aeruginosa* on *C. albicans*, hyphal development was the most annotated VM (**Figure 5**), for which the majority of interactions (75%) were inhibitory. Concerning the most reported source entities affecting hyphae, most annotated interactions reported effects of bacteria as a whole (annotated as “cell”), meaning that no molecular entity was identified/tested. LPS, rhamnolipids, and 3-oxo-C12-HSL were also annotated as inhibiting hyphal growth (Hall et al., 2011; Bandara et al., 2013; Watrous et al., 2013), while HHQ and PQS had no effect (Reen et al., 2011; Watrous et al., 2013). Biofilm formation by *C. albicans* was the second most annotated affected VM as result of *P. aeruginosa* interaction. In this case, almost all interactions were inhibitory (93%) and caused by different source entities (**Figure 5**). No VF were annotated for *C. albicans*.

Regarding the effect of *C. albicans* on *P. aeruginosa*, swarming motility, adhesion, and biofilm formation were the most annotated VM (**Figure 5**). Swarming motility is inhibited in the presence of *C. albicans* due to farnesol (McAlester et al., 2008) and ethanol (Chen et al., 2014). The adhesion capability of *P. aeruginosa* was annotated as inhibited by farnesol (McAlester et al., 2008) and stimulated by ethanol (Chen et al., 2014). In the case of biofilm formation, 50% of interactions were of stimulation and the other 50% had a null effect. Concerning the source entities, these distinct biofilm effects were both annotated for the entity “cell”, while only stimulation was annotated for ethanol (**Figure 5**).

Pyocyanin production by *P. aeruginosa* was the most annotated VF (**Figure 5**) and the effect of *C. albicans* on this molecule apparently depends on the mode of growth, with stimulation in biofilm growth and inhibition in planktonic growth. These differences are further explored in a later section. Other annotated VF includes pyoverdine, proteases, rhamnolipids, and haemolysin (**Figure 5**). All interactions annotated for pyoverdine and rhamnolipids were stimulatory, with “cell” as the source entity and biofilm as the mode of growth for the first and both planktonic and biofilm as modes of growth for the latter. Both AI of *C. albicans*, farnesol and tyrosol, were annotated as inhibiting haemolysin in *P. aeruginosa* in planktonic cultures (Abdel-Rhman et al., 2015). Regarding the production of proteases, tyrosol inhibited these enzymes, while a null effect was annotated for farnesol, in the planktonic mode of growth (Abdel-Rhman et al., 2015).

### Interaction Effects on Gene and Protein Expression

**Figure 6:**
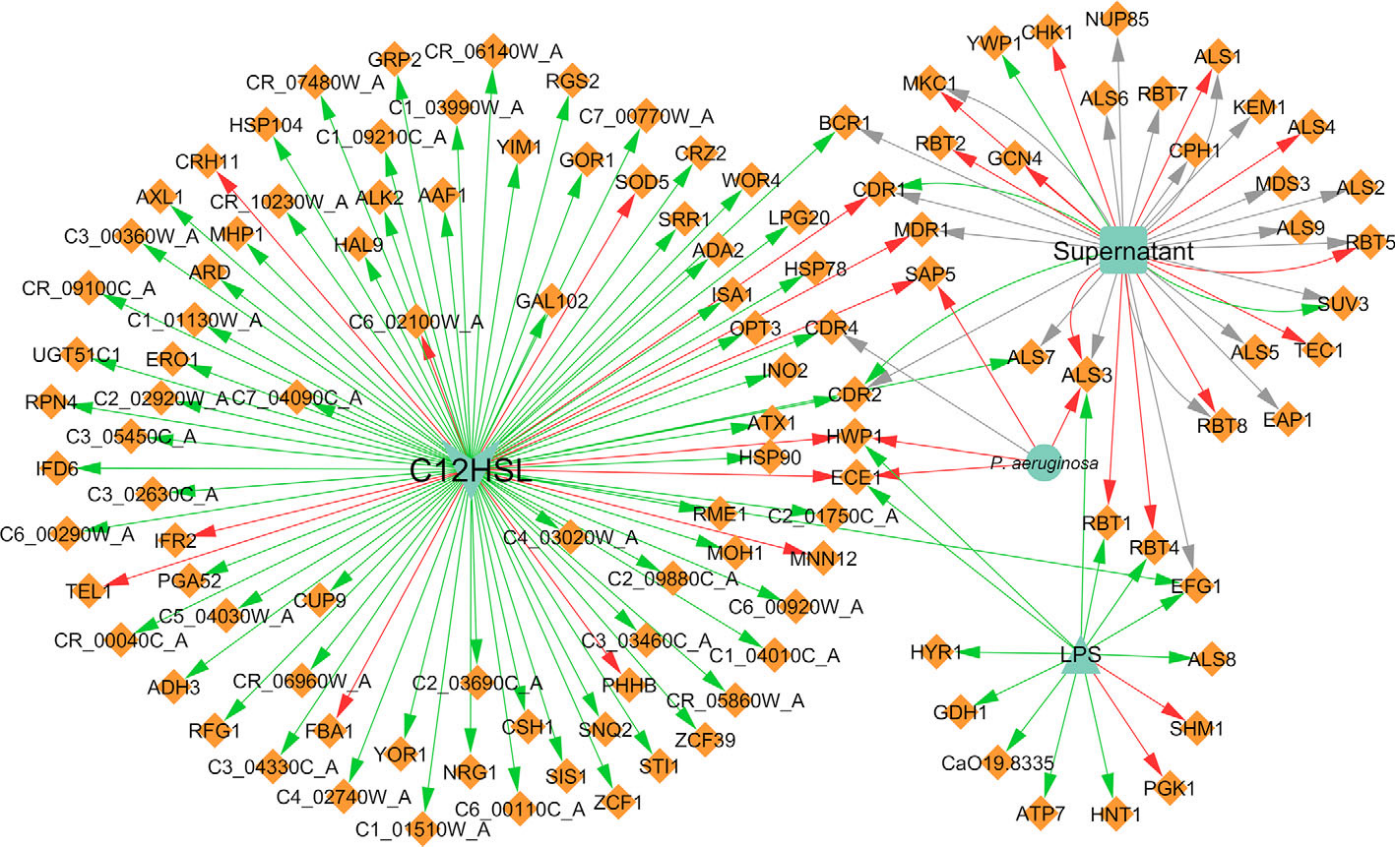
Network of the effects of *P. aeruginosa* on *C. albicans* gene expression. Legend: Teal nodes, *P. aeruginosa*; orange nodes, *C. albicans*; green arrows, upregulation; grey arrows, null effect; red arrows: downregulation; node and node label sizes are directly proportional to the number of related (outward and inward) edges (interactions).

Concerning the expression of genes and proteins as result of microbial interaction, it was possible to annotate a total of 110 distinct genes differentially expressed in *C. albicans* due to the presence of *P. aeruginosa*, of which 83 were upregulated, 21 were downregulated, and six were both up and downregulated (**Figure 6**). These findings are correlated with the affected VM previously mentioned, especially hyphal growth. For instance, *HWP1* and *ECE1*, along with *ALS3*, all hyphae-specific genes, were downregulated in the presence of 3-oxo-C12-HSL (Hogan et al., 2004) and of *P. aeruginosa* “cell” (Park et al., 2014) and its supernatant (Holcombe et al., 2010). These results are in accordance with the well-known inhibitory effect annotated for hyphal growth. However, there are some cases where some contradictory effects were annotated for other source entities, namely LPS upregulation of the previously mentioned genes (Bandara et al., 2013). This and other contrasting annotations are discussed in the next section.

**Figure 7:**
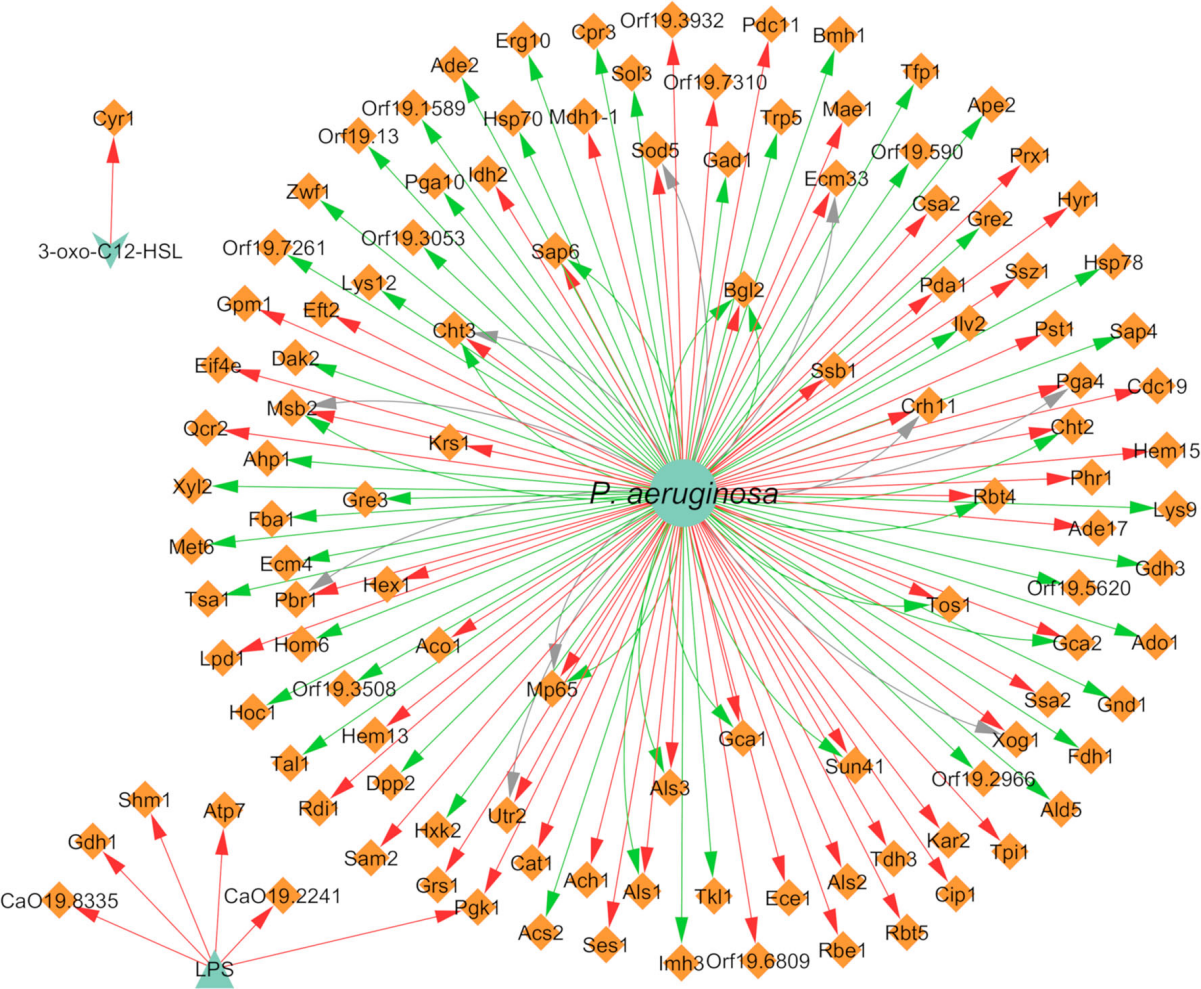
Network of the effects of *P. aeruginosa* on *C. albicans* protein expression. Legend: Teal nodes, *P. aeruginosa*; orange nodes, *C. albicans*; green arrows, upregulation; grey arrows, null effect; red arrows: downregulation; node and node label sizes are directly proportional to the number of related (outward and inward) edges (interactions).

With concern to protein expression, *C. albicans* differentially expressed 117 proteins, of which 59 were annotated as upregulated and 69 as downregulated (**Figure 7**). Some proteins are reported in more than one paper or even with different interaction types in the same paper; hence, there is a greater number of annotated interactions (**Figure 7**) in relation to the total number of different annotated proteins. Almost all interactions with proteins had “cell” as the source entity. Protein expression was also shown to vary depending on the time of maturation of the dual-species biofilm. For instance, proteins related with adhesion and biofilm formation, namely Als1, Als2, Als3, and Pbr1, seem to be negatively affected by the presence of *P. aeruginosa* in a time dependent manner, being less expressed in later stages of biofilm development. The differences observed throughout time can be related with the interaction between both pathogens that probably is more pronounced with increased time of interaction, correlating with the lowering of the metabolic activity of *C. albicans* in the double consortia (Purschke et al., 2012).

Three different proteins related to virulence in *C. albicans*, namely Tfp1, Ape2, and Bgl2, were annotated as upregulated in the presence of *P. aeruginosa* (Trejo-Hernández et al., 2014). This upregulation could have a synergistic interaction with the VF of the bacterium, resulting in enhanced pathogenesis. All proteins of the cell wall were annotated as downregulated after interaction of the fungus with *P. aeruginosa*. This includes the cell wall proteins Crh11 and Ecm33, involved in cell wall assembly and regeneration, filamentation, and adherence to host cells, and also the hyphal cell wall proteins Rbt5, Hyr1, Ece1, and Rbe1 (Purschke et al., 2012).

**Figure 8:**
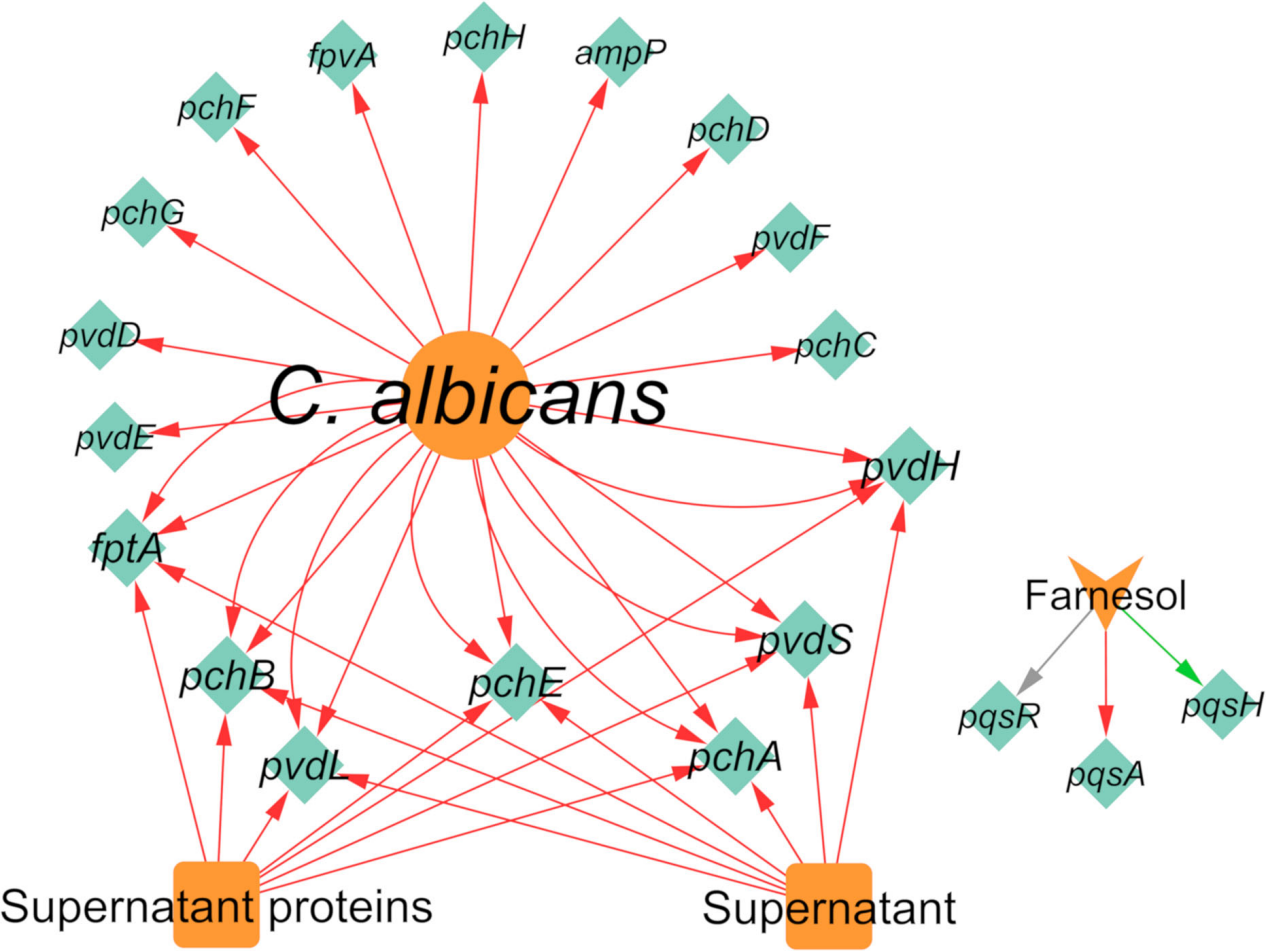
Network of the effects of *C. albicans* on *P. aeruginosa* gene expression. Legend: Teal nodes, *P. aeruginosa*; orange nodes, *C. albicans*; green arrows, upregulation; grey arrows, null effect; red arrows: downregulation; node and node label sizes are directly proportional to the number of related (outward and inward) edges (interactions).

Regarding the effect of *C. albicans* on the gene expression of *P. aeruginosa*, 19 differentially expressed genes were annotated, of which 18 were downregulated and one was upregulated (**Figure 8**). It was very interesting to note that almost all interactions were of downregulation (90%). In fact, *pqsH* was the only gene of *P. aeruginosa* annotated as being upregulated in the presence of farnesol (Cugini et al., 2007). This QS-related gene is involved in the terminal step of the biosynthesis of quinolones by catalyzing the hydroxylation of HHQ to PQS (Déziel et al., 2004). Most of the downregulated annotated genes belong to the *pvd* and *pch* gene families (**Figure 8**) in planktonic and *in vivo* conditions. Interestingly, the related protein PchD was upregulated in biofilm conditions (Purschke et al., 2012; Trejo-Hernández et al., 2014). These and other contrasting annotations are discussed in the next section.

**Figure 9:**
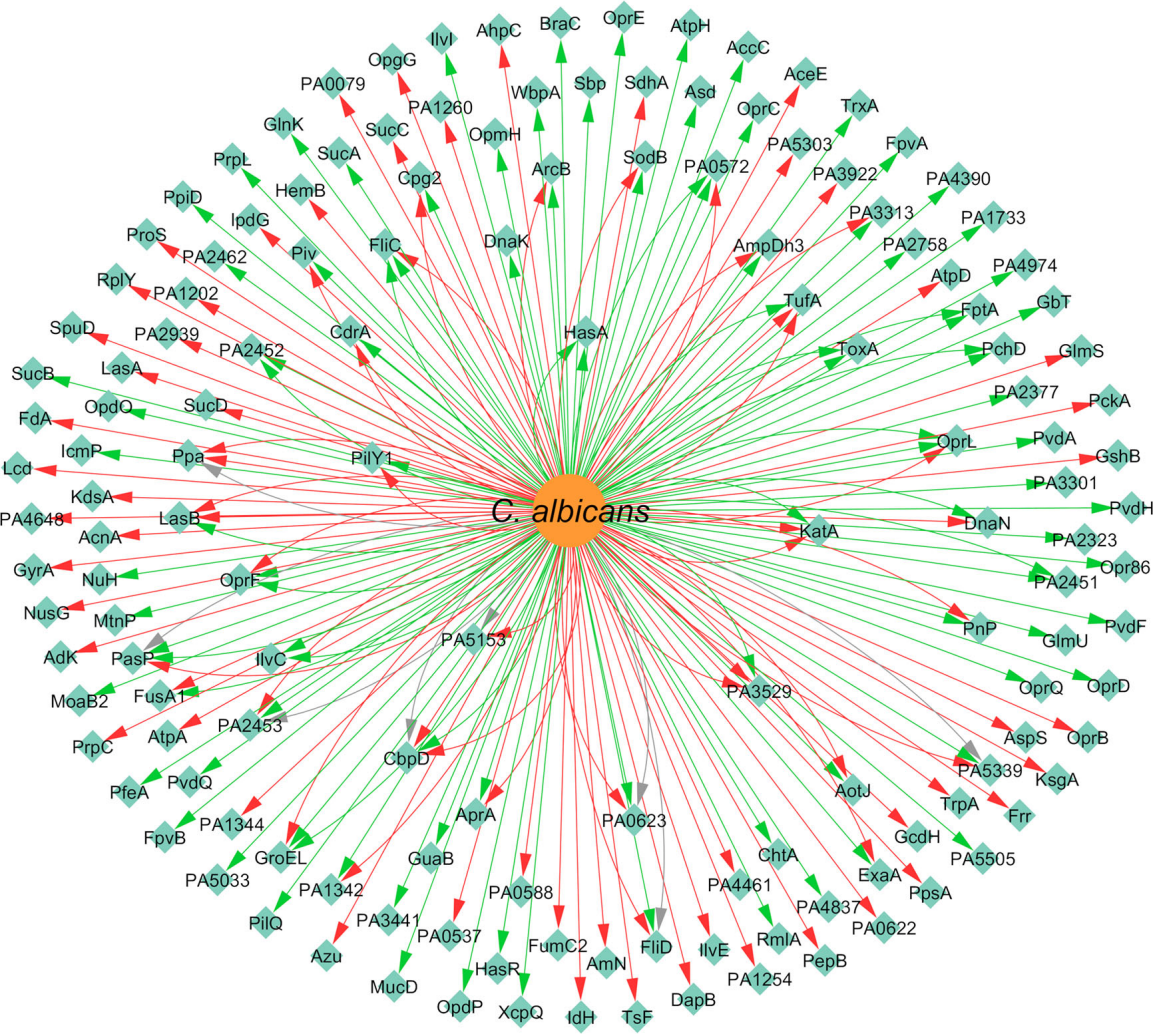
Network of the effects of *C. albicans* on *P. aeruginosa* protein expression. Legend: Teal nodes, *P. aeruginosa*; orange nodes, *C. albicans*; green arrows, upregulation; grey arrows, null effect; red arrows: downregulation; node and node label sizes are directly proportional to the number of related (outward and inward) edges (interactions).

Concerning the expression of proteins, *P. aeruginosa* differentially expressed 147 proteins due to the presence of *C. albicans*. A total of 92 of these proteins were upregulated and 86 were downregulated (**Figure 9**). The majority of the annotated proteins from *P. aeruginosa* related to virulence were upregulated due to the interaction with *C. albicans*. For instance, proteins related to siderophore biosynthesis and/or transport, namely ChtA, FptA, FpvA, PchD, FpvB, PvdA, PvdH, PvdF, and PvdQ, were all upregulated in biofilm settings (Purschke et al., 2012; Trejo-Hernández et al., 2014). Other upregulated proteins, namely OpdO, OpdP OpmH, Opr86, OprC, OprE, and OprQ, are involved in the transport of small molecules and antibiotic resistance (Trejo-Hernández et al., 2014). HasA and HasR, two proteins related to heme uptake, were also upregulated as well as PilQ and XcpQ, which are proteins responsible for motility and attachment of the bacterium (Purschke et al., 2012; Trejo-Hernández et al., 2014). Proteins involved in cell wall and LPS synthesis, namely, GlmU, RmlA, and WbpA, were also annotated as upregulated. These findings reinforce the notion that *P. aeruginosa* becomes more virulent as consequence of the interaction with *C. albicans*.

### Contrasting Annotations Illustrating the Complexity of Interspecies Study

Upon the analysis of all the gathered information, it was possible to discern some seemingly opposite effects annotated from the literature regarding *P. aeruginosa*-*C. albicans* interactions. All of these annotated interactions are outlined in **Tables 2** and **3** for *P. aeruginosa* > *C. albicans* and *C. albicans* > *P. aeruginosa* interactions, respectively. These tables contain not only information on the interactions but also on the experimental conditions in which they were observed, as these define their comparability.

**Table 2:** Contrasting *P. aeruginosa* > *C. albicans* interactions.

Target	Interaction	Source	Mode of growth	Experimental Conditions*		Method	Strains		Reference
				Time (h)	Media		PA	CA	
Als1	Upregulation	Cell	Biofilm	1.5, 4.5, 6	YNBNP	MALDI-TOF MS/MS	PAO1	SC5314	(Purschke et al., 2012)
	Downregulation			3, 24, 48					
*ALS1*	Downregulation	Supernatant		24 + 6 (treat.)		Microarray	HSL non-producing isolates (CF177, PAO1ΔQS)		(Holcombe et al., 2010)
Als3	Downregulation	Cell	Biofilm	6, 24, 48	YNBNP	MALDI-TOF MS/MS	PAO1	SC5314	(Purschke et al., 2012)
	Upregulation			1.5, 3, 4.5					
*ALS3*	Downregulation	Cell		24	TSB	RT-qPCR	Clinical isolate	Clinical isolate	(Park et al., 2014)
		Supernatant		24 + 6 (treat.)	YNBNP	Microarray, RT-PCR	HSL producing (PAO1, CF144) and non-producing (CF177, PAO1ΔQS) isolates	SC5314	(Holcombe et al., 2010)
	Upregulation	LPS		48	YNB	RT-qPCR	ATCC 27316	SC5314	(Bandara et al., 2013)
Atp7	Downregulation	LPS	Biofilm	18	YNB	2D-PAGE; MALDI-TOF	ATCC 27316	SC5314	(Bandara et al., 2013)
*ATP7*	Upregulation			48		RT-qPCR			
Bgl2	Downregulation	Cell	Biofilm	3, 4.5, 6, 24, 48	YNBNP	MALDI-TOF MS/MS	PAO1	SC5314	(Purschke et al., 2012)
	Upregulation			1.5					
	Upregulation			24	RPMI 1640 + L-glutamine + dextrose + uridine	2D-PAGE; MALDI-TOF		CAI4	(Trejo-Hernández et al., 2014)
*CDR1*	Downregulation	3-oxo-C12-HSL	Biofilm	24 + 1 (treat.)	YNB + glucose	RT-qPCR	(n.a.)	SC5314	(Bandara et al., 2020)
	Upregulation	Supernatant		24 + 6 (treat.)	YNBNP	Microarray	PAO1		(Holcombe et al., 2010)
Cht2	Downregulation	Cell	Biofilm	3, 4.5, 6, 24, 48	YNBNP	MALDI-TOF MS/MS	PAO1	CAI4	(Purschke et al., 2012)
	Upregulation			1.5					
Cht3	Downregulation	Cell	Biofilm	4.5, 6, 24, 48	YNBNP	MALDI-TOF MS/MS	PAO1	CAI4	(Purschke et al., 2012)
	Upregulation			3					
Ece1	Downregulation	Cell	Biofilm	1.5, 3, 4.5, 6	YNBNP	MALDI-TOF MS/MS	PAO1	SC5314	(Purschke et al., 2012)
*ECE1*	Downregulation	Cell	Biofilm	24	TSB	RT-qPCR	Clinical isolate	Clinical isolate	(Park et al., 2014)
		3-oxo-C12-HSL	Planktonic	8	YNBNP		PA14	SC5314	(Hogan et al., 2004)
	Upregulation	LPS	Biofilm	48	YNB		ATCC 27316	SC5314	(Bandara et al., 2013)
Fba1	Upregulation	Cell	Biofilm	24	RPMI 1640 + L-glutamine + dextrose + uridine	2D-PAGE; MALDI-TOF	PAO1	CAI4	(Trejo-Hernández et al., 2014)
*FBA1*	Downregulation	3-oxo-C12-HSL			YNB + glucose	RNA-Seq.	(n.a.)	SC5314	(Bandara et al., 2020)
Gca1	Downregulation	Cell	Biofilm	3, 4.5, 6, 48	YNBNP	MALDI-TOF MS/MS	PAO1	SC5314	(Purschke et al., 2012)
	Upregulation			24					
Gca2	Downregulation	Cell	Biofilm	3, 4.5, 6, 48	YNBNP	MALDI-TOF MS/MS	PAO1	SC5314	(Purschke et al., 2012)
	Upregulation			24					
Gdh1	Downregulation	LPS	Biofilm	48	YNB	2D-PAGE; MALDI-TOF	ATCC 27316	SC5314	(Bandara et al., 2013)
*GDH1*	Upregulation					RT-qPCR			
*HWP1*	Downregulation	Cell	Biofilm	24	TSB	RT-qPCR	Clinical isolate	Clinical isolate	(Park et al., 2014)
		3-oxo-C12-HSL	Planktonic	8	YNBNP		PA14	SC5314	(Hogan et al., 2004)
	Upregulation	LPS	Biofilm	48	YNB		ATCC 27316	SC5314	(Bandara et al., 2013)
Hyr1	Downregulation	Cell	Biofilm	48	YNBNP	MALDI-TOF MS/MS	PAO1	SC5314	(Purschke et al., 2012)
*HYR1*	Upregulation	LPS			YNB	RT-qPCR	ATCC 27316		(Bandara et al., 2013)
Mp65	Downregulation	Cell	Biofilm	48	YNBNP	MALDI-TOF MS/MS	PAO1	SC5314	(Purschke et al., 2012)
	Upregulation			1.5, 3, 4.5, 6					
Msb2	Downregulation	Cell	Biofilm	6	YNBNP	MALDI-TOF MS/MS	PAO1	SC5314	(Purschke et al., 2012)
	Upregulation			3, 24, 48					
*RBT1*	Downregulation	Supernatant	Biofilm	24 + 6 (treat.)	YNBNP	Microarray, RT-PCR	HSL producing (PAO1, CF144) and non-producing (CF177, PAO1ΔQS) isolates	SC5314	(Holcombe et al., 2010)
	Upregulation	LPS		48	YNB	RT-qPCR	ATCC 27316		(Bandara et al., 2013)
Rbt4	Downregulation	Cell	Biofilm	3, 4.5, 6, 24, 48	YNBNP	MALDI-TOF MS/MS	PAO1	SC5314	(Purschke et al., 2012)
	Upregulation			1.5					
*RBT4*	Upregulation	LPS		48	YNB	RT-qPCR	ATCC 27316		(Bandara et al., 2013)
Sap6	Downregulation	Cell	Biofilm	24, 48	YNBNP	MALDI-TOF MS/MS	PAO1	SC5314	(Purschke et al., 2012)
	Upregulation			3, 4.5, 6					
Sun41	Downregulation	Cell	Biofilm	24, 48	YNBNP	MALDI-TOF MS/MS	PAO1	SC5314	(Purschke et al., 2012)
	Upregulation			3, 4.5, 6					
Tos1	Downregulation	Cell	Biofilm	24, 48	YNBNP	MALDI-TOF MS/MS	PAO1	SC5314	(Purschke et al., 2012)
	Upregulation			3, 4.5, 6					

PA, P. aeruginosa; CA, C. albicans; CF, Cystic Fibrosis; MALDI-TOF, Matrix-Assisted Laser Desorption/Ionization, Time of Flight; MS/MS, tandem Mass Spectrometry; qPCR, quantitative Polymerase Chain Reaction; RNA-Seq., RNA Sequencing; RT-PCR, Reverse Transcription Polymerase Chain Reaction; 2D-PAGE, two-Dimensional PolyAcrylamide Gel Electrophoresis; YNBNP, Yeast Nitrogen Base N-acetyl-D-glucosamine Phosphate; TSB, Tryptic Soy Broth; treat., treatment; n.a., not applicable. *all experiments were conducted at 37^o^C.

**Table 3:** Contrasting *C. albicans* > *P. aeruginosa* interactions.

Target	Interaction	Source	Mode of growth	Experimental Conditions*		Method	Strains		Reference
				Time (h)	Media		PA	CA	
AmpDh3	Downregulation	Cell	Biofilm	1.5, 4.5, 6, 24, 48	YNBNP	MALDI-TOF MS/MS	PAO1	SC5314	(Purschke et al., 2012)
	Upregulation			3					
AprA	Downregulation	Cell	Biofilm	1.5, 3, 4.5, 6, 24, 48	YNBNP	MALDI-TOF MS/MS	PAO1	SC5314	(Purschke et al., 2012)
	Upregulation			24	RPMI 1640 + L-glutamine + dextrose + uridine	2D-PAGE; MALDI-TOF		CAI4	(Trejo-Hernández et al., 2014)
ArcB	Downregulation	Cell	Biofilm	1.5, 24, 48	YNBNP	MALDI-TOF MS/MS	PAO1	SC5314	(Purschke et al., 2012)
	Upregulation			3					
CbpD	Downregulation	Cell	Biofilm	24	RPMI 1640 + L-glutamine + dextrose + uridine	2D-PAGE; MALDI-TOF	PAO1	CAI4	(Trejo-Hernández et al., 2014)
	Downregulation			1.5, 48	YNBNP	MALDI-TOF MS/MS		SC5314	(Purschke et al., 2012)
	Upregulation			3, 6, 24					
CdrA	Downregulation	Cell	Biofilm	24,48	YNBNP	MALDI-TOF MS/MS	PAO1	SC5314	(Purschke et al., 2012)
	Upregulation			3, 4.5, 6					
Cpg2	Downregulation	Cell	Biofilm	4.5	YNBNP	MALDI-TOF MS/MS	PAO1	SC5314	(Purschke et al., 2012)
	Upregulation			48					
DnaN	Downregulation	Cell	Biofilm	24	RPMI 1640 + L-glutamine + dextrose + uridine	2D-PAGE; MALDI-TOF	PAO1	CAI4	(Trejo-Hernández et al., 2014)
	Upregulation			48	YNBNP	MALDI-TOF MS/MS		SC5314	(Purschke et al., 2012)
ExaA	Downregulation	Cell	Biofilm	24	YNBNP	MALDI-TOF MS/MS	PAO1	SC5314	(Purschke et al., 2012)
	Upregulation			48					
FliC	Upregulation	Cell	Biofilm	24	RPMI 1640 + L-glutamine + dextrose + uridine	2D-PAGE; MALDI-TOF	PAO1	CAI4	(Trejo-Hernández et al., 2014)
	Upregulation			4.5	YNBNP	MALDI-TOF MS/MS		SC5314	(Purschke et al., 2012)
	Downregulation			1.5, 3, 24,48					
FliD	Downregulation	Cell	Biofilm	1.5, 6, 24, 48	YNBNP	MALDI-TOF MS/MS	PAO1	SC5314	(Purschke et al., 2012)
	Upregulation			3					
FptA	Upregulation	Cell	Biofilm	24	RPMI 1640 + L-glutamine + dextrose + uridine	2D-PAGE; MALDI-TOF	PAO1	CAI4	(Trejo-Hernández et al., 2014)
*fptA*	Downregulation	Cell	*In vivo*	(n.a.)	Virulence murine model	RNA-Seq.	PAO1	SC5314	(Lopez-Medina et al., 2015)
			Planktonic	10 min	GGP + YPD	RT-qPCR			
		Supernatant	Planktonic	10 min	GGP				
		Supernatant proteins		10 min	GGP				
FpvA	Upregulation	Cell	Biofilm	48	YNBNP	MALDI-TOF MS/MS	PAO1	SC5314	(Purschke et al., 2012)
				24	RPMI 1640 + L-glutamine + dextrose + uridine	2D-PAGE; MALDI-TOF		CAI4	(Trejo-Hernández et al., 2014)
*fpvA*	Downregulation	Cell	*In vivo*	(n.a.)	Virulence murine model	RNA-Seq.		SC5314	(Lopez-Medina et al., 2015)
FusA1	Downregulation	Cell	Biofilm	24	RPMI 1640 + L-glutamine + dextrose + uridine	2D-PAGE; MALDI-TOF	PAO1	CAI4	(Trejo-Hernández et al., 2014)
	Upregulation			48	YNBNP	MALDI-TOF MS/MS		SC5314	(Purschke et al., 2012)
GroEL	Upregulation	Cell	Biofilm	24	RPMI 1640 + L-glutamine + dextrose + uridine	2D-PAGE; MALDI-TOF	PAO1	CAI4	(Trejo-Hernández et al., 2014)
	Upregulation			6	YNBNP	MALDI-TOF MS/MS		SC5314	(Purschke et al., 2012)
	Downregulation			1.5, 24, 48					
KatA	Downregulation	Cell	Biofilm	24	RPMI 1640 + L-glutamine + dextrose + uridine	2D-PAGE; MALDI-TOF	PAO1	CAI4	(Trejo-Hernández et al., 2014)
	Downregulation			1.5, 4.5, 6, 24, 48	YNBNP	MALDI-TOF MS/MS		SC5314	(Purschke et al., 2012)
	Upregulation			3					
LasB	Downregulation	Cell	Biofilm	24	RPMI 1640 + L-glutamine + dextrose + uridine	2D-PAGE; MALDI-TOF	PAO1	CAI4	(Trejo-Hernández et al., 2014)
	Downregulation			1.5, 4.5, 6	YNBNP	MALDI-TOF MS/MS		SC5314	(Purschke et al., 2012)
	Upregulation			3, 24					
OprF	Upregulation	Cell	Biofilm	24	RPMI 1640 + L-glutamine + dextrose + uridine	2D-PAGE; MALDI-TOF	PAO1	CAI4	(Trejo-Hernández et al., 2014)
	Upregulation			3	YNBNP	MALDI-TOF MS/MS		SC5314	(Purschke et al., 2012)
	Downregulation			1.5, 4.5, 6, 24, 48					
OprL	Upregulation	Cell	Biofilm	24	RPMI 1640 + L-glutamine + dextrose + uridine	2D-PAGE; MALDI-TOF	PAO1	CAI4	(Trejo-Hernández et al., 2014)
	Upregulation			6	YNBNP	MALDI-TOF MS/MS		SC5314	(Purschke et al., 2012)
	Downregulation			1.5, 4.5, 24, 48					
PA0572	Upregulation	Cell	Biofilm	24	RPMI 1640 + L-glutamine + dextrose + uridine	2D-PAGE; MALDI-TOF	PAO1	CAI4	(Trejo-Hernández et al., 2014)
	Upregulation			3, 4.5, 6, 24, 48	YNBNP	MALDI-TOF MS/MS		SC5314	(Purschke et al., 2012)
	Downregulation			1.5					
PA0623	Downregulation	Cell	Biofilm	24, 48	YNBNP	MALDI-TOF MS/MS	PAO1	SC5314	(Purschke et al., 2012)
	Upregulation			3, 6					
PA1342	Downregulation	Cell	Biofilm	1.5, 4.5, 6, 24, 48	YNBNP	MALDI-TOF MS/MS	PAO1	SC5314	(Purschke et al., 2012)
	Upregulation			3					
PA2453	Downregulation	Cell	Biofilm	24, 48	YNBNP	MALDI-TOF MS/MS	PAO1	SC5314	(Purschke et al., 2012)
	Upregulation			1.5, 4.5, 6					
PA3313	Downregulation	Cell	Biofilm	24	YNBNP	MALDI-TOF MS/MS	PAO1	SC5314	(Purschke et al., 2012)
	Upregulation			48					
PA3529	Downregulation	Cell	Biofilm	24	RPMI 1640 + L-glutamine + dextrose + uridine	2D-PAGE; MALDI-TOF	PAO1	CAI4	(Trejo-Hernández et al., 2014)
	Downregulation			24	YNBNP	MALDI-TOF MS/MS	PAO1	SC5314	(Purschke et al., 2012)
	Upregulation			48					
PA5339	Downregulation	Cell	Biofilm	1.5, 4.5, 6	YNBNP	MALDI-TOF MS/MS	PAO1	SC5314	(Purschke et al., 2012)
	Upregulation			48					
PasP	Upregulation	Cell	Biofilm	24	RPMI 1640 + L-glutamine + dextrose + uridine	2D-PAGE; MALDI-TOF	PAO1	CAI4	(Trejo-Hernández et al., 2014)
	Upregulation			3, 24, 48	YNBNP	MALDI-TOF MS/MS		SC5314	(Purschke et al., 2012)
	Downregulation			1.5, 4.5					
PchD	Upregulation	Cell	Biofilm	1.5	YNBNP	MALDI-TOF MS/MS	PAO1	SC5314	(Purschke et al., 2012)
	Upregulation			24	RPMI 1640 + L-glutamine + dextrose + uridine	2D-PAGE; MALDI-TOF		CAI4	(Trejo-Hernández et al., 2014)
*pchD*	Downregulation		*In vivo*	(n.a.)	Virulence murine model	RNA-Seq.		SC5314	(Lopez-Medina et al., 2015)
PilY1	Downregulation	Cell	Biofilm	48	YNBNP	MALDI-TOF MS/MS	PAO1	SC5314	(Purschke et al., 2012)
	Upregulation			24					
Piv	Downregulation	Cell	Biofilm	1.5, 3, 4.5, 48	YNBNP	MALDI-TOF MS/MS	PAO1	SC5314	(Purschke et al., 2012)
	Upregulation			6, 24					
PnP (extracellular)	Downregulation	Cell	Biofilm	24	RPMI 1640 + L-glutamine + dextrose + uridine	2D-PAGE; MALDI-TOF	PAO1	CAI4	(Trejo-Hernández et al., 2014)
PnP (intracellular)	Upregulation								
PQS	Stimulation	Farnesol	Planktonic	14	LB agar	TLC	PA14, PA14Δ*lasR*, PA14Δ*lasI*	(n.a.)	(Cugini et al., 2010)
	Stimulation	Cell		24, 96 (stirred batch)	ASM	Bioluminescence (reporter strain PAO1 ΔpqsA CTX-lux::pqsA)	PAO1	SC5314	(O’Brien and Welch, 2019)
	Inhibition	Farnesol		6, 24	LB	TLC	PA14	(n.a.)	(Cugini et al., 2007)
*pqsH*	Upregulation	Farnesol	Planktonic	14	LB agar	RT-qPCR	PA14Δ*lasR*+pUCP22, PA14Δ*lasR*+pPQSHPA14Δ*pqsH*+pUCP22, PA14Δ*pqsH*+pPQSH	SC5314	(Cugini et al., 2010)
*pqsA*	Downregulation	Farnesol	Planktonic	15 min	LB	RT-PCR	PA14	(n.a.)	(Cugini et al., 2007)
PvdF	Upregulation	Cell	Biofilm	24	RPMI 1640 + L-glutamine + dextrose + uridine	2D-PAGE; MALDI-TOF	PAO1	CAI4	(Trejo-Hernández et al., 2014)
*pvdF*	Downregulation		*In vivo*	(n.a.)	Virulence murine model	RNA-Seq.		SC5314	(Lopez-Medina et al., 2015)
PvdH	Upregulation	Cell	Biofilm	24	RPMI 1640 + L-glutamine + dextrose + uridine	2D-PAGE; MALDI-TOF	PAO1	CAI4	(Trejo-Hernández et al., 2014)
*pvdH*	Downregulation	Cell	*In vivo*	(n.a.)	Virulence murine model	RNA-Seq.		SC5314	(Lopez-Medina et al., 2015)
			Planktonic	10 min	GGP + YPD	RT-qPCR			
		Supernatant	Planktonic	10 min	GGP	RT-qPCR			
		Supernatant proteins							
Pyocyanin	Stimulation	Cell	Biofilm	24	RPMI 1640 + L-glutamine + dextrose + uridine	HPLC	PAO1	CAI4	(Trejo-Hernández et al., 2014)
		Farnesol	Planktonic	14	LB agar	Chloroform extraction; Spectrophotometry	PA14Δ*lasR*, PA14Δ*lasI*	SC5314	(Cugini et al., 2010)
	Inhibition	Cell	Planktonic	96 (aerobic batch and stirred batch)	ASM	Chloroform extraction; Spectrophotometry	PAO1	SC5314	(O’Brien and Welch, 2019)
		Farnesol	Planktonic	6, 24	LB		PA14, PAO1	(n.a.)	(Cugini et al., 2007)
SodB	Downregulation	Cell	Biofilm	1.5, 4.5, 24, 48	YNBNP	MALDI-TOF MS/MS	PAO1	SC5314	(Purschke et al., 2012)
	Upregulation			3					
TufA	Downregulation	Cell	Biofilm	24	RPMI 1640 + L-glutamine + dextrose + uridine	2D-PAGE; MALDI-TOF	PAO1	CAI4	(Trejo-Hernández et al., 2014)
	Downregulation			1.5	YNBNP	MALDI-TOF MS/MS		SC5314	(Purschke et al., 2012)
	Upregulation			48					

PA, P. aeruginosa; CA, C. albicans; MALDI-TOF, Matrix-Assisted Laser Desorption/Ionization, Time of Flight; MS/MS, tandem Mass Spectrometry; qPCR, quantitative Polymerase Chain Reaction; RNA-Seq., RNA Sequencing; RT-PCR, Reverse Transcription Polymerase Chain Reaction; 2D-PAGE, two-Dimensional PolyAcrylamide Gel Electrophoresis; ASM, Artificial Sputum Medium; GGP, Glucose-Glycerol-Peptone; RPMI, Roswell Park Memorial Institute Medium; YNBNP, Yeast Nitrogen Base N-acetyl-D-glucosamine Phosphate; n.a., not applicable. *all experiments were conducted at 37^o^C.

One of the more noticeable factors leading to contrasting observations is the period of time during which cells are co-cultivated or exposed to each other’s molecular factors (e.g. AI, supernatants). For example, Purschke and colleagues showed the time-dependent differential expression of several *P. aeruginosa* and *C. albicans* proteins when the two microorganisms are grown together as mixed biofilms (Purschke et al., 2012). Many of these proteins are up- or downregulated dependent if cells are harvested at early or late time points (or vice-versa), which is corroborated by some similar observations in other publications (**Tables 2**, **3**). However, this is not always the case. For instance, Als3, a hyphae specific protein of *C. albicans*, appears to be upregulated at early and downregulated at later time-points when in contact with *P. aeruginosa* or its supernatant (Holcombe et al., 2010; Purschke et al., 2012; Park et al., 2014). Still, *ALS3* was shown to be upregulated by LPS at 48 h (Bandara et al., 2013) (**Table 2**). The effect of LPS is concentration and strain dependent (Bandara et al., 2013), which could explain the differences observed.

Another important factor is the effector entity at play. Although most papers, as previously mentioned, use the whole bacterial cell to investigate its effect onto the other co-existing species, many studies use single molecular players to try to pinpoint the exact mechanisms behind the observed interactions. However, even when one molecular player seems to correlate with the effect of the whole cell, others sometimes seem to contradict it. For example, the gene *CDR1* encodes a drug efflux pump protein that is upregulated by the supernatant of *P. aeruginosa* PAO1 (Holcombe et al., 2010) but downregulated by 3-oxo-C12-HSL in *C. albicans* biofilms (Bandara et al., 2020) (**Table 2**). Although 3-oxo-C12-HSL is extracellular, therefore usually present in *P. aeruginosa*’s supernatants, this contrasting observation is likely due to other metabolites present in the supernatant perceived as detrimental by *Candida*, which, therefore, activates efflux pumps to extrude them (Holcombe et al., 2010).

The type of media and, as consequence, the mode of growth are also key factors to take into consideration when analyzing interspecies studies. Cugini et al demonstrated that farnesol inhibits PQS in a liquid *P. aeruginosa* culture (Cugini et al., 2007), but stimulates PQS production when *P. aeruginosa* is grown in solid media (Cugini et al., 2010). Virulence related genes, namely *fptA*, *fpvA*, *pchD*, *pvdF*, and *pvdH* were upregulated in *P. aeruginosa* biofilms (Purschke et al., 2012; Trejo-Hernández et al., 2014) but downregulated in planktonic cultures and in an *in vivo* murine model of a gastro-intestinal (GI) infection (Lopez-Medina et al., 2015) (**Table 3**). In the murine GI tract, *P*. *aeruginosa* is present in two populations: i) in the gut lumen and ii) adherent to the epithelium, in which only a subset may be in biofilms (Lopez-Medina et al., 2015).

It is safe to say that the comparability of the observations made in interspecies studies is highly, if not totally, dependent on the experimental conditions, which should mimic, as far as possible, the real-like conditions of infections. The standardization of experiments and methodologies should be a priority in order to more quickly advance this research area.

### Alternative Approaches in *P. aeruginosa*-*C. albicans* Biofilm Control: QS Inhibition

Biofilm is known as the preferred mode of growth of most microorganisms, including bacterial and fungal pathogens. These consortia are typically resilient with dynamic structures, giving their microbial constituents a broad range of advantages, as previously mentioned. In real infection scenarios, microorganisms are usually found in the biofilm form and these consortia can easily turn an infection into a chronic condition (Bjarnsholt, 2013). Thus, although planktonic testing is practical and informative, studies involving biofilms can better mimic a real-life infection scenario and allow a better comprehension about the microbial behavior under these situations. Concerning the annotated information in this work, although the number of annotated interactions was higher for biofilms (83%), this only reflects the types of methods being used. Actually, the number of studies using planktonic or biofilm as the mode of growth was similar (1518 *vs* 1417, respectively), which shows that biofilm studies are still lacking in order to get a real perspective on these inter-species interactions.

Given the biofilm problematic, along with the ever-rising antimicrobial resistance, the need for alternative therapies is in high demand. For example, antivirulence agents carry advantages like circumvention of antibiotic resistance, by targeting VF rather than bacterial growth (Totsika, 2016), and a high number of putative virulent targets (Allen et al., 2014). Considering that QS is the main regulator of virulence in both bacteria and fungi, the use of quorum quenching (QQ) compounds that inhibit specific QS mechanisms related to virulence can be a promising strategy to modulate it (Chan et al., 2015). Additionally, QQ compounds are probably less likely to induce resistance in cases where their targets are located extracellularly (Fetzner, 2015). Notwithstanding all the advantages, target selection in this approach is of critical importance given the existence of redundancy and alternative regulatory pathways, which may compensate for a given disturbance, and the complexity of the outcomes of inter-species interactions, which can make an apparently detrimental effect on the pathogen lead to an opposite desired effect in the infection as a whole. For example, the interference with the QS system of one pathogen can potentially facilitate the pathogenicity of the other co-infecting species. Moreover, effective anti-virulence therapy would probably entail combinations with other agents, such as other antivirulence drugs or even antibiotics, to increase antimicrobial effectiveness in polymicrobial communities (Magalhães et al., 2019). Other factors to take into account when designing anti-QS approaches is the negative impacts on the host and its microflora, the possibility of bacteremia/sepsis as a consequence of the biofilm disruption, altered immune and inflammatory responses, and resistance development for intracellular targets (Krzyżek, 2019)

As far as it was possible to check, there are no studies regarding the use of anti-QS or anti-virulence compounds against polymicrobial biofilms of *P. aeruginosa* and *C. albicans*. However, some interesting works have been published recently for each pathogen alone. Works prior to 2017 for *P. aeruginosa* and to 2018 for *C. albicans* can be consulted in the publications by Pérez-Pérez et al. (2017) and Grainha et al. (2018), respectively. Particularly, some focus has been given to anti-QS agents targeting the major regulator PqsR. This QS system of *P. aeruginosa*, related to the AI PQS and to HHQ, plays a major role in biofilm formation and inflammation, being crucial in chronic infection establishment. Such agents include synthetic compounds, such as quinolone- and quinazolinone-based derivatives, that compete and/or antagonize this QS system, but also natural compounds (Soheili et al., 2019). Concerning their impact on co-infection scenarios, not much information was annotated for the effect of this system and its AI on *C. albicans*. HHQ was annotated as inhibiting *C. albicans* biofilm formation in a concentration dependent-manner (Reen et al., 2011), which means that disruption of HHQ action could be detrimental for *P. aeruginosa* but positive for the fungus.

The idea of using the AI themselves to control virulence and biofilm formation has also been tested. There are many studies using exogenous farnesol to control biofilms of *C. albicans* (Grainha et al., 2018). A recent study used both farnesol and tyrosol to control *C. albicans* biofilms in hopes of using them as adjuvant in oral hygiene (Sebaa et al., 2019). In fact, farnesol was annotated as affecting the production of AI from *P. aeruginosa*, namely PQS, HHQ, and C4-HSL, and their related genes, depending on the mode of growth. The upregulation of the expression of AI and of the VF pyocyanin by farnesol in *P. aeruginosa* in biofilm settings (Cugini et al., 2010) has to be evaluated concerning the effect of this molecule if used to treat mixed consortia.

## Conclusions

It is well known that *P. aeruginosa* and *C. albicans* coexist in many infections, but, unfortunately, the mechanisms of bacterial-fungal interactions remain unclear. A better understanding of the social behavior in the consortium and how microbes change their virulence as result of established interactions is clinically relevant, being crucial to comprehend the infection process while simultaneously guiding new effective therapeutic strategies. Bacteria and fungi influence each other directly or indirectly in different ways and this work clearly illustrated the amount and complexity of the mechanisms behind such interactions. Importantly, it was interesting to observe that the interaction type was dependent on experimental conditions, namely the mode of growth.

This work allowed the deposition of all available information on the scientific literature regarding *P. aeruginosa* and *C. albicans* interactions on an existing database (www.ceb.uminho.pt/ISCTD) which will assist research on this topic by cutting on effort and time spent tracking down and analyzing the scientific literature. As future work, a continuous update of this knowledge database will be performed, with additional information of new discoveries and new microorganisms.

## Data Availability Statement

Publicly available datasets were analyzed in this study. This data can be found here: https://www.ceb.uminho.pt/ISCTD/.

## Author Contributions

TG and PJ are equally contributing authors. TG, PJ, and MP designed the experiments. TG performed the annotation of the information. TG and PJ analyzed the data. TG, PJ, DA, and SL wrote the manuscript. All authors contributed to the article and approved the submitted version.

## Funding

This work was supported by the Portuguese Foundation for Science and Technology (FCT) under the scope of the strategic funding of UID/BIO/04469/2020 unit and BioTecNorte operation (NORTE-01-0145-FEDER-000004) funded by the European Regional Development Fund under the scope of Norte2020–Programa Operacional Regional do Norte. The authors also acknowledge COMPETE2020 and FCT for the project POCI-01-0145-FEDER-029841 and FCT for the PhD Grant of TG [grant number SFRH/BD/136544/2018].

## Conflict of Interest

The authors declare that the research was conducted in the absence of any commercial or financial relationships that could be construed as a potential conflict of interest.

## Supplementary Material

The Supplementary Material for this article canbe found online at: https://www.frontiersin.org/articles/10.3389/fcimb.2020.550505/full#supplementary-material

## References

[B1] Abdel-Rhman S. H. El-Mahdy A. M. El-Mow M. (2015). Effect of Tyrosol and Farnesol on Virulence and Antibiotic Resistance of Clinical Isolates of Pseudomonas aeruginosa. BioMed. Res. Int. 2015,7. 10.1155/2015/456463 PMC4710896 26844228

[B2] Ader F. Jawhara S. Nseir S. Kipnis E. Faure K. Vuotto F. (2011). Short term Candida albicans colonization reduces Pseudomonas aeruginosa-related lung injury and bacterial burden in a murine model. Crit. Care 15, R150. 10.1186/cc10276 21689424 PMC3219024

[B3] Alem M. A. S. Oteef M. D. Y. Flowers T. H. Douglas L. J. (2006). Production of tyrosol by Candida albicans biofilms and its role in quorum sensing and biofilm development. Eukaryot. Cell 5, 1770–1779. 10.1128/EC.00219-06 16980403 PMC1595342

[B4] Allen R. C. Popat R. Diggle S. P. Brown S. P. (2014). Targeting virulence: can we make evolution-proof drugs? Nat. Rev. Microbiol. 12, 300–308. 10.1038/nrmicro3232 24625893

[B5] Allesen-Holm M. Barken K. B. Yang L. Klausen M. Webb J. S. Kjelleberg S. (2006). A characterization of DNA release in *Pseudomonas aeruginosa* cultures and biofilms. Mol. Microbiol 59, 1114–1128. 10.1111/j.1365-2958.2005.05008.x 16430688

[B6] Auger P. Joly J. (1977). Factors influencing germ tube production in Candida albicans. Mycopathologia 61, 183–186. 10.1007/BF00468014 22042

[B7] Azam M. W. Khan A. U. (2019). Updates on the pathogenicity status of Pseudomonas aeruginosa. Drug Discovery Today 24, 350–359. 10.1016/j.drudis.2018.07.003 30036575

[B8] Bai F. Cai Z. Yang L. (2019). Recent progress in experimental and human disease-associated multi-species biofilms. Comput. Struct. Biotechnol. J. 17, 1234–1244. 10.1016/j.csbj.2019.09.010 31921390 PMC6944735

[B9] Bandara H. M. Yau J. Y. Watt R. M. Jin L. J. Samaranayake L. P. (2010). Pseudomonas aeruginosa inhibits in-vitro Candida biofilm development. BMC Microbiol. 10, 125. 10.1186/1471-2180-10-125 20416106 PMC2874548

[B10] Bandara H. M. H. N. Cheung B. P. K. Watt R. M. Jin L. J. Samaranayake L. P. (2013). Pseudomonas aeruginosa lipopolysaccharide inhibits Candida albicans hyphae formation and alters gene expression during biofilm development. Mol. Oral. Microbiol. 28, 54–69. 10.1111/omi.12006 23194472

[B11] Bandara H. M. H. N. Wood D. L. A. Vanwonterghem I. Hugenholtz P. Cheung B. P. K. Samaranayake L. P. (2020). Fluconazole resistance in Candida albicans is induced by Pseudomonas aeruginosa quorum sensing. Sci. Rep. 10, 7769. 10.1038/s41598-020-64761-3 32385378 PMC7211000

[B12] Bjarnsholt T. (2013). The role of bacterial biofilms in chronic infections. APMIS. Suppl. 121, 1–51. 10.1111/apm.12099 23635385

[B13] Brand A. Barnes J. D. Mackenzie K. S. Odds F. C. Gow N. A. R. (2008). Cell wall glycans and soluble factors determine the interactions between the hyphae of Candida albicans and Pseudomonas aeruginosa. FEMS Microbiol. Lett. 287, 48–55. 10.1111/j.1574-6968.2008.01301.x 18680523 PMC2613227

[B14] Burrows L. L. (2018). The Therapeutic Pipeline for Pseudomonas aeruginosa Infections. ACS Infect. Dis. 4, 1041–1047. 10.1021/acsinfecdis.8b00112 29771109

[B15] Calderone R. A. Fonzi W. A. (2001). Virulence factors of Candida albicans. Trends Microbiol. 9, 327–335. 10.1016/S0966-842X(01)02094-7 11435107

[B16] Chan K.-G. Liu Y.-C. Chang C.-Y. (2015). Inhibiting N-acyl-homoserine lactone synthesis and quenching *Pseudomonas* quinolone quorum sensing to attenuate virulence. Front. Microbiol. 6, 1173. 10.3389/fmicb.2015.01173 26539190 PMC4609879

[B17] Chen H. Fink G. R. (2006). Feedback control of morphogenesis in fungi by aromatic alcohols. Genes Dev. 20, 1150–1161. 10.1101/gad.1411806 16618799 PMC1472474

[B18] Chen H. Fujita M. Feng Q. Clardy J. Fink G. R. (2004). Tyrosol is a quorum-sensing molecule in Candida albicans. Proc. Natl. Acad. Sci. U. S. A. 101, 5048–5052. 10.1073/pnas.0401416101 15051880 PMC387371

[B19] Chen A. II Dolben E. F. Okegbe C. Harty C. E. Golub Y. Thao S. (2014). Candida albicans Ethanol Stimulates Pseudomonas aeruginosa WspR-Controlled Biofilm Formation as Part of a Cyclic Relationship Involving Phenazines. PloS Pathog. 10, e1004480. 10.1371/journal.ppat.1004480 25340349 PMC4207824

[B20] Clarivate (2020). Web of Science [v.5.35] - Web of Science Core Collection Basic Search. Available at: https://apps.webofknowledge.com/WOS_GeneralSearch_input.do?product=WOS&search_mode=GeneralSearch&SID=F6pmkX9oPVd3x6VKfyp&preferencesSaved (Accessed June 9, 2020).

[B21] Coenye T. Nelis H. J. (2010). In vitro and in vivo model systems to study microbial biofilm formation. J. Microbiol. Methods 83, 89–105. 10.1016/j.mimet.2010.08.018 20816706

[B22] Cugini C. Calfee M. W. Farrow J. M. Morales D. K. Pesci E. C. Hogan D. A. (2007). Farnesol, a common sesquiterpene, inhibits PQS production in Pseudomonas aeruginosa. Mol. Microbiol. 65, 896–906. 10.1111/j.1365-2958.2007.05840.x 17640272

[B23] Cugini C. Morales D. K. Hogan D. A. (2010). Candida albicans-produced farnesol stimulates Pseudomonas quinolone signal production in LasR-defective Pseudomonas aeruginosa strains. Microbiology 156, 3096–3107. 10.1099/mic.0.037911-0 20656785 PMC3068698

[B24] Curutiu C. Ditu L. M. Iordache F. Bleotu C. Chifiriuc M. C. Lazar V. (2017). Quorum Sensing molecules produced by Pseudomonas aeruginosa impair attachment and biofilm formation in Candida albicans. Biointerface Res. Appl. Chem. 7, 2016–2020.

[B25] Dadar M. Tiwari R. Karthik K. Chakraborty S. Shahali Y. Dhama K. (2018). Candida albicans - Biology, molecular characterization, pathogenicity, and advances in diagnosis and control – An update. Microb. Pathog. 117, 128–138. 10.1016/j.micpath.2018.02.028 29454824

[B26] Davis-Hanna A. Piispanen A. E. Stateva L. II Hogan D. A. (2007). Farnesol and dodecanol effects on the Candida albicans Ras1-cAMP signalling pathway and the regulation of morphogenesis. Mol. Microbiol. 67, 47–62. 10.1111/j.1365-2958.2007.06013.x PMC3782305 18078440

[B27] De Vos M. G. J. Zagorski M. McNally A. Bollenbach T. (2017). Interaction networks, ecological stability, and collective antibiotic tolerance in polymicrobial infections. Proc. Natl. Acad. Sci. U. S. A. 114, 10666–10671. 10.1073/pnas.1713372114 28923953 PMC5635929

[B28] Desai J. V. (2018). Candida albicans hyphae: From growth initiation to invasion. J. Fungi 4, 10. 10.3390/jof4010010 PMC5872313 29371503

[B29] Déziel E. Lépine F. Milot S. He J. Mindrinos M. N. Tompkins R. G. (2004). Analysis of Pseudomonas aeruginosa 4-hydroxy-2-alkylquinolines (HAQs) reveals a role for 4-hydroxy-2-heptylquinoline in cell-to-cell communication. Proc. Natl. Acad. Sci. U.S.A. 101, 1339–1344. 10.1073/pnas.0307694100 14739337 PMC337054

[B30] Dhamgaye S. Qu Y. Peleg A. Y. (2016). Polymicrobial infections involving clinically relevant Gram-negative bacteria and fungi. Cell. Microbiol. 18, 1716–1722. 10.1111/cmi.12674 27665610

[B31] Diaz P. II Strausbaugh L. D. Dongari-Bagtzoglou A. (2014). Fungal-bacterial interactions and their relevance to oral health: Linking the clinic and the bench. Front. Cell. Infect. Microbiol. 4, 101. 10.3389/fcimb.2014.00101 25120959 PMC4114182

[B32] Diggle S. P. Matthijs S. Wright V. J. Fletcher M. P. Chhabra S. R. Lamont I. L. (2007). The *Pseudomonas aeruginosa* 4-quinolone signal molecules HHQ and PQS play multifunctional roles in quorum sensing and iron entrapment. Chem. Biol. 14, 87–96. 10.1016/j.chembiol.2006.11.014 17254955

[B33] Dixon E. F. Hall R. A. (2015). Noisy neighbourhoods: quorum sensing in fungal-polymicrobial infections. Cell. Microbiol. 17, 1431–1441. 10.1111/cmi.12490 26243526 PMC4973845

[B34] Dižová S. Bujdáková H. (2017). Properties and role of the quorum sensing molecule Farnesol in relation to the yeast Candida albicans. Pharmazie 72, 307–312. 10.1691/ph.2017.6174 29442016

[B35] Fetzner S. (2015). Quorum quenching enzymes. J. Biotechnol. 201, 2–14. 10.1016/j.jbiotec.2014.09.001 25220028

[B36] Flemming H.-C. Wingender J. (2010). The biofilm matrix. Nat. Rev. Microbiol. 8, 623–633. 10.1038/nrmicro2415 20676145

[B37] Flemming H.-C. Wingender J. Szewzyk U. Steinberg P. Rice S. A. Kjelleberg S. (2016). Biofilms: an emergent form of bacterial life. Nat. Rev. Microbiol. 14, 563–575. 10.1038/nrmicro.2016.94 27510863

[B38] Fourie R. Pohl C. H. (2019). Beyond antagonism: The interaction between Candida species and Pseudomonas aeruginosa. J. Fungi 5, 34. 10.3390/jof5020034 PMC6617365 31010211

[B39] Fourie R. Ells R. Swart C. W. Sebolai O. M. Albertyn J. Pohl C. H. (2016). Candida albicans and Pseudomonas aeruginosa interaction, with focus on the role of eicosanoids. Front. Physiol. 7, 64. 10.3389/fphys.2016.00064 26955357 PMC4767902

[B40] Fourie R. Ells R. Kemp G. Sebolai O. M. Albertyn J. Pohl C. H. (2017). Pseudomonas aeruginosa produces aspirin insensitive eicosanoids and contributes to the eicosanoid profile of polymicrobial biofilms with Candida albicans. Prostagland. Leukot. Essent. Fat. Acids 117, 36–46. 10.1016/j.plefa.2017.01.008 28237086

[B41] Fuqua W. C. Winans S. C. Greenberg E. P. (1994). Quorum sensing in bacteria: the LuxR-LuxI family of cell density-responsive transcriptional regulators. J. Bacteriol. 176, 269–275. 10.1128/JB.176.2.269-275.1994 8288518 PMC205046

[B42] Gambello M. J. Kaye S. Iglewski B. H. (1993). LasR of Pseudomonas aeruginosa is a transcriptional activator of the alkaline protease gene (apr) and an enhancer of exotoxin A expression. Infect. Immun. 61, 1180–1184. 10.1128/iai.61.4.1180-1184.1993 8454322 PMC281346

[B43] Gellatly S. L. Hancock R. E. W. (2013). *Pseudomonas aeruginosa*: new insights into pathogenesis and host defenses. Pathog. Dis. 67, 159–173. 10.1111/2049-632X.12033 23620179

[B44] Gibson J. Sood A. Hogan D. A. (2009). Pseudomonas aeruginosa-Candida albicans interactions: localization and fungal toxicity of a phenazine derivative. Appl. Environ. Microbiol. 75, 504–513. 10.1128/AEM.01037-08 19011064 PMC2620721

[B45] Gloyne L. S. Grant G. D. Perkins A. V. Powell K. L. McDermott C. M. Johnson P. V. (2011). Pyocyanin-induced toxicity in A549 respiratory cells is causally linked to oxidative stress. Toxicol. Vitr. 25, 1353–1358. 10.1016/j.tiv.2011.05.004 21596130

[B46] Grahl N. Demers E. G. Lindsay A. K. Harty C. E. Willger S. D. Piispanen A. E. (2015). Mitochondrial Activity and Cyr1 Are Key Regulators of Ras1 Activation of C. albicans Virulence Pathways. PloS Pathog. 11, e1005133. 10.1371/journal.ppat.1005133 26317337 PMC4552728

[B47] Grainha T. R. R. Jorge P. A. S. Pérez-Pérez M. Rodríguez G. P. Pereira M. O. B. O. Lourenço A. M. G. (2018). Exploring anti-quorum sensing and anti-virulence based strategies to fight Candida albicans infections: An in silico approach. FEMS Yeast Res. 18, foy022. 10.1093/femsyr/foy022 29518242

[B48] Hall R. A. Turner K. J. Chaloupka J. Cottier F. De Sordi L. Sanglard D. (2011). The quorum-sensing molecules farnesol/homoserine lactone and dodecanol operate via distinct modes of action in Candida albicans. Eukaryot. Cell 10, 1034–1042. 10.1128/EC.05060-11 21666074 PMC3165441

[B49] Hamet M. Pavon A. Dalle F. Pechinot A. Prin S. Quenot J. P. (2012). Candida spp. airway colonization could promote antibiotic-resistant bacteria selection in patients with suspected ventilator-associated pneumonia. Intensive Care Med. 38, 1272–1279. 10.1007/s00134-012-2584-2 22699790

[B50] Han T. L. Cannon R. D. Villas-Bôas S. G. (2011). The metabolic basis of Candida albicans morphogenesis and quorum sensing. Fungal Genet. Biol. 48, 747–763. 10.1016/j.fgb.2011.04.002 21513811

[B51] Harriott M. M. Noverr M. C. (2011). Importance of Candida-bacterial polymicrobial biofilms in disease. Trends Microbiol. 19, 557–563. 10.1016/j.tim.2011.07.004 21855346 PMC3205277

[B52] Hauser A. R. (2011). Pseudomonas aeruginosa: So many virulence factors, so little time. Crit. Care Med. 39, 2193–2194. 10.1097/CCM.0b013e318221742d 21849835 PMC3359648

[B53] Hawver L. A. Jung S. A. Ng W.-L. (2016). Specificity and complexity in bacterial quorum-sensing systems. FEMS Microbiol. Rev. 40, 738–752. 10.1093/femsre/fuw014 27354348 PMC5007282

[B54] Hogan D. A. Kolter R. (2002). Pseudomonas-Candida interactions: an ecological role for virulence factors. Science 296, 2229–2232. 10.1126/science.1070784 12077418

[B55] Hogan D. A. Vik A. Kolter R. (2004). A Pseudomonas aeruginosa quorum-sensing molecule influences Candida albicans morphology. Mol. Microbiol. 54, 1212–1223. 10.1111/j.1365-2958.2004.04349.x 15554963

[B56] Holcombe L. J. McAlester G. Munro C. A. Enjalbert B. Brown A. J. P. Gow N. A. R. (2010). Pseudomonas aeruginosa secreted factors impair biofilm development in Candida albicans. Microbiology 156, 1476–1485. 10.1099/mic.0.037549-0 20150241

[B57] Hornby J. M. Jensen E. C. Lisec A. D. Tasto J. J. Jahnke B. Shoemaker R. (2001). Quorum Sensing in the Dimorphic Fungus Candida albicans Is Mediated by Farnesol. Appl. Environ. Microbiol. 67, 2982–2992. 10.1128/AEM.67.7.2982-2992.2001 11425711 PMC92970

[B58] Hughes W. T. Kim H. K. (1973). Mycoflora in cystic fibrosis: Some ecologic aspects of pseudomonas aeruginosa and Candida albicans. Mycopathol. Mycol. Appl. 50, 261–269. 10.1007/BF02053377 4199669

[B59] Jakobsen T. H. Bjarnsholt T. Jensen P. Ø. Givskov M. Høiby N. (2013). Targeting quorum sensing in *Pseudomonas aeruginosa* biofilms: current and emerging inhibitors. Future Microbiol. 8, 901–921. 10.2217/fmb.13.57 23841636

[B60] Jimenez P. N. Koch G. Thompson J. A. Xavier K. B. Cool R. H. Quax W. J. (2012). The multiple signaling systems regulating virulence in Pseudomonas aeruginosa. Microbiol. Mol. Biol. Rev. 76, 46–65. 10.1128/MMBR.05007-11 22390972 PMC3294424

[B61] Jones S. Yu B. Bainton N. J. Birdsall M. Bycroft B. W. Chhabra S. R. (1993). The lux autoinducer regulates the production of exoenzyme virulence determinants in Erwinia carotovora and Pseudomonas aeruginosa. EMBO J. 12, 2477–2482. 10.1002/j.1460-2075.1993.tb05902.x 8508773 PMC413484

[B62] Kaleli I. Cevahir N. Demir M. Yildirim U. Sahin R. (2007). Anticandidal activity of Pseudomonas aeruginosa strains isolated from clinical specimens. Mycoses 50, 74–78. 10.1111/j.1439-0507.2006.01322.x 17302753

[B63] Kerr J. R. Taylor G. W. Rutman A. Høiby N. Cole P. J. Wilson R. (1999). Pseudomonas aeruginosa pyocyanin and 1-hydroxyphenazine inhibit fungal growth. J. Clin. Pathol. 52, 385–387. 10.1136/jcp.52.5.385 10560362 PMC1023078

[B64] Khan M. S. A. Alshehrei F. Al-Ghamdi S. B. Bamaga M. A. Al-Thubiani A. S. Alam M. Z. (2020). Virulence and biofilms as promising targets in developing antipathogenic drugs against candidiasis. Futur. Sci. OA 6, FSO440. 10.2144/fsoa-2019-0027 PMC6997914 32025329

[B65] Krzyżek P. (2019). Challenges and limitations of anti-quorum sensing therapies. Front. Microbiol 10, 2473. 10.3389/fmicb.2019.02473 31736912 PMC6834643

[B66] Kumar S. N. Nisha G. Sudaresan A. Venugopal V. Kumar M. S. Lankalapalli R. (2014). Synergistic Activity of Phenazines Isolated From Pseudomonas Aeruginosa in Combination With Azoles Against Candida Species. Med. Mycol. 52, 482–490. 10.1093/MMY/MYU012 24915852

[B67] Kumar A. Alam A. Rani M. Ehtesham N. Z. Hasnain S. E. (2017). Biofilms: Survival and defense strategy for pathogens. Int. J. Med. Microbiol. 307, 481–489. 10.1016/J.IJMM.2017.09.016 28950999

[B68] Lebeaux D. Chauhan A. Rendueles O. Beloin C. (2013). From in vitro to in vivo models of bacterial biofilm-related infections. Pathogens 2, 288–356. 10.3390/pathogens2020288 25437038 PMC4235718

[B69] Lee J. Zhang L. (2015). The hierarchy quorum sensing network in Pseudomonas aeruginosa. Protein Cell 6, 26–41. 10.1007/s13238-014-0100-x 25249263 PMC4286720

[B70] Lewis K. A. Baker A. E. Chen A. II Harty C. E. Kuchma S. L. O’Toole G. A. (2019). Ethanol decreases pseudomonas aeruginosa flagellar motility through the regulation of flagellar stators. J. Bacteriol. (American Soc. Microbiol.) 201, e00285–19. 10.1128/JB.00285-19 PMC6707923 31109994

[B71] Lin C. J. Wu C. Y. Yu S. J. Chen Y. L. (2018). Protein kinase A governs growth and virulence in Candida tropicalis. Virulence 9, 331–347. 10.1080/21505594.2017.1414132 29254431 PMC5955175

[B72] Lindsay A. K. Deveau A. Piispanen A. E. Hogan D. A. (2012). Farnesol and cyclic AMP signaling effects on the hypha-to-yeast transition in Candida albicans. Eukaryot. Cell 11, 1219–1225. 10.1128/EC.00144-12 22886999 PMC3485915

[B73] Lindsay A. K. Morales D. K. Liu Z. Grahl N. Zhang A. Willger S. D. (2014). Analysis of Candida albicans Mutants Defective in the Cdk8 Module of Mediator Reveal Links between Metabolism and Biofilm Formation. PloS Genet. 10, e1004567. 10.1371/journal.pgen.1004567 25275466 PMC4183431

[B74] Lingappa B. T. Prasad M. Lingappa Y. Hunt D. F. Biemann K. (1969). Phenethyl alcohol and tryptophol: autoantibiotics produced by the fungus Candida albicans. Science 163, 192–194. 10.1126/science.163.3863.192 5762768

[B75] Lopez-Medina E. Fan D. Coughlin L. A. Ho E. X. Lamont I. L. Reimmann C. (2015). Candida albicans Inhibits Pseudomonas aeruginosa Virulence through Suppression of Pyochelin and Pyoverdine Biosynthesis. PloS Pathog. 11, e1005129. 10.1371/journal.ppat.1005129 26313907 PMC4552174

[B76] Magalhães A. P. Jorge P. Pereira M. O. (2019). *Pseudomonas aeruginosa* and *Staphylococcus aureus* communication in biofilm infections: insights through network and database construction. Crit. Rev. Microbiol. 45, 712–728. 10.1080/1040841X.2019.1700209 31835971

[B77] Maza P. K. Bonfim-Melo A. Padovan A. C. B. Mortara R. A. Orikaza C. M. Ramos L. M. D. (2017). Candida albicans: The Ability to Invade Epithelial Cells and Survive under Oxidative Stress Is Unlinked to Hyphal Length. Front. Microbiol. 8, 1235. 10.3389/fmicb.2017.01235 28769876 PMC5511855

[B78] McAlester G. O’Gara F. Morrissey J. P. (2008). Signal-mediated interactions between Pseudomonas aeruginosa and Candida albicans. J. Med. Microbiol. 57, 563–569. 10.1099/jmm.0.47705-0 18436588

[B79] Méar J.-B. Kipnis E. Faure E. Dessein R. Schurtz G. Faure K. (2013). Candida albicans and Pseudomonas aeruginosa interactions: more than an opportunistic criminal association? Méd. Mal. Infect. 43, 146–151. 10.1016/j.medmal.2013.02.005 23622953

[B80] Moradali M. F. Ghods S. Rehm B. H. A. (2017). Pseudomonas aeruginosa lifestyle: A paradigm for adaptation, survival, and persistence. Front. Cell. Infect. Microbiol. 7, 39. 10.3389/fcimb.2017.00039 28261568 PMC5310132

[B81] Morales D. K. Hogan D. A. (2010). Candida albicans interactions with bacteria in the context of human health and disease. PloS Pathog. 6, e1000886. 10.1371/journal.ppat.1000886 20442787 PMC2861711

[B82] Morales D. K. Grahl N. Okegbe C. Dietrich L. E. P. Jacobs N. J. Hogan D. A. (2013). Control of Candida albicans metabolism and biofilm formation by Pseudomonas aeruginosa phenazines. MBio 4, e00526–12. 10.1128/mBio.00526-12 PMC3560528 23362320

[B83] Mulcahy L. R. Isabella V. M. Lewis K. (2014). Pseudomonas aeruginosa Biofilms in Disease. Microb. Ecol 18, 1–12. 10.1007/s00248-013-0297-x PMC3977026 24096885

[B84] NCBI (2004). PubMed [Internet]. Bethesda Natl. Libr. Med. (US), Natl. Cent. Biotechnol. Inf. Available at: https://pubmed.ncbi.nlm.nih.gov/ (Accessed February 14, 2020).

[B85] Nogueira F. Sharghi S. Kuchler K. Lion T. (2019). Pathogenetic impact of bacterial–fungal interactions. Microorganisms 7, 459. 10.3390/microorganisms7100459 PMC6843596 31623187

[B86] Oh K.-B. Miyazawa H. Naito T. Matsuoka H. (2001). Purification and characterization of an autoregulatory substance capable of regulating the morphological transition in Candida albicans. Proc. Natl. Acad. Sci. 98, 4664–4668. 10.1073/pnas.071404698 11274356 PMC31891

[B87] O’Brien T. J. Welch M. (2019). A Continuous-Flow Model for in vitro Cultivation of Mixed Microbial Populations Associated With Cystic Fibrosis Airway Infections. Front. Microbiol. 10, 2713. 10.3389/fmicb.2019.02713 31824471 PMC6883238

[B88] O’Malley Y. Q. Abdalla M. Y. McCormick M. L. Reszka K. J. Denning G. M. Britigan B. E. (2003). Subcellular localization of Pseudomonas pyocyanin cytotoxicity in human lung epithelial cells. Am. J. Physiol. - Lung Cell. Mol. Physiol. 284, L420–L430. 10.1152/ajplung.00316.2002 12414438

[B89] Ovchinnikova E. S. Krom B. P. Van Der Mei H. C. Busscher H. J. (2012). Force microscopic and thermodynamic analysis of the adhesion between Pseudomonas aeruginosa and Candida albicans. Soft Matter 8, 6454–6461. 10.1039/c2sm25100k

[B90] Pang Z. Raudonis R. Glick B. R. Lin T. J. Cheng Z. (2019). Antibiotic resistance in *Pseudomonas aeruginosa*: Mechanisms and alternative therapeutic strategies. Biotechnol. Adv. 37, 177–192. 10.1016/j.biotechadv.2018.11.013 30500353

[B91] Park S. J. Han K. H. Park J. Y. Choi S. J. Lee K. H. (2014). Influence of bacterial presence on biofilm formation of Candida albicans. Yonsei Med. J. 55, 449–458. 10.3349/ymj.2014.55.2.449 24532517 PMC3936627

[B92] Passador L. Cook J. M. Gambello M. J. Rust L. Iglewski B. H. (1993). Expression of Pseudomonas aeruginosa virulence genes requires cell-to-cell communication. Science 260, 1127–1130. 10.1126/science.8493556 8493556

[B93] Paul D. Gopal J. Kumar M. Manikandan M. (2018). Nature to the natural rescue: Silencing microbial chats. Chem. Biol. Interact. 280, 86–98. 10.1016/j.cbi.2017.12.018 29247642

[B94] Pearson J. P. Passador L. Iglewski B. H. Greenberg E. P. (1995). A second N-acylhomoserine lactone signal produced by Pseudomonas aeruginosa. Proc. Natl. Acad. Sci. U. S. A. 92, 1490–1494. 10.1073/PNAS.92.5.1490 7878006 PMC42545

[B95] Peleg A. Y. Hogan D. A. Mylonakis E. (2010). Medically important bacterialg-fungal interactions. Nat. Rev. Microbiol. 8, 340–349. 10.1038/nrmicro2313 20348933

[B96] Pena R. T. Blasco L. Ambroa A. González-Pedrajo B. Fernández-García L. López M. (2019). Relationship between quorum sensing and secretion systems. Front. Microbiol. 10, 1100. 10.3389/fmicb.2019.01100 31231316 PMC6567927

[B97] Pérez-Pérez M. Jorge P. Pérez Rodríguez G. Pereira M. O. Lourenço A. (2017). Quorum sensing inhibition in *Pseudomonas aeruginosa* biofilms: new insights through network mining. Biofouling 33, 128–142. 10.1080/08927014.2016.1272104 28121162

[B98] Pestrak M. J. Chaney S. B. Eggleston H. C. Dellos-Nolan S. Dixit S. Mathew-Steiner S. S. (2018). Pseudomonas aeruginosa rugose small-colony variants evade host clearance, are hyper-inflammatory, and persist in multiple host environments. PloS Pathog. 14, e1006842. 10.1371/journal.ppat.1006842 29394295 PMC5812653

[B99] Peters B. M. Jabra-Rizk M. A. O’May G. A. Costerton J. W. Shirtliff M. E. (2012). Polymicrobial interactions: Impact on pathogenesis and human disease. Clin. Microbiol. Rev. 25, 193–213. 10.1128/CMR.00013-11 22232376 PMC3255964

[B100] Phelan V. V. Moree W. J. Aguilar J. Cornett D. S. Koumoutsi A. Noble S. M. (2014). Impact of a transposon insertion in phzF2 on the specialized metabolite production and interkingdom interactions of Pseudomonas aeruginosa. J. Bacteriol. 196, 1683–1693. 10.1128/JB.01258-13 24532776 PMC3993319

[B101] Polke M. Jacobsen I. D. (2017). Quorum sensing by farnesol revisited. Curr. Genet 63, 791–797. 10.1007/s00294-017-0683-x 28247023

[B102] Potera C. (2014). Researchers Find Surprises in Human Microbiome. BioScience 64, 760–765. 10.1093/biosci/biu122

[B103] Poulain D. (2015). Candida albicans, plasticity and pathogenesis. Crit. Rev. Microbiol. 41, 208–217. 10.3109/1040841X.2013.813904 23962107

[B104] Purschke F. G. Hiller E. Trick I. Rupp S. (2012). Flexible survival strategies of Pseudomonas aeruginosa in biofilms result in increased fitness compared with Candida albicans. Mol. Cell. Proteomics 11, 1652–1669. 10.1074/mcp.M112.017673 22942357 PMC3518115

[B105] Reen F. J. Mooij M. J. Holcombe L. J. Mcsweeney C. M. Mcglacken G. P. Morrissey J. P. (2011). The Pseudomonas quinolone signal (PQS), and its precursor HHQ, modulate interspecies and interkingdom behaviour. FEMS Microbiol. Ecol. 77, 413–428. 10.1111/j.1574-6941.2011.01121.x 21539583

[B106] Rodrigues M. E. Lopes S. P. Pereira C. R. Azevedo N. F. Lourenço A. Henriques M. (2017). Polymicrobial ventilator-associated pneumonia: Fighting in vitro Candida albicans-pseudomonas aeruginosa biofilms with antifungal-antibacterial combination therapy. PloS One 12, e0170433. 10.1371/journal.pone.0170433 28114348 PMC5256963

[B107] Roux D. Gaudry S. Khoy-Ear L. Aloulou M. Phillips-Houlbracq M. Bex J. (2013). Airway fungal colonization compromises the immune system allowing bacterial pneumonia to prevail. Crit. Care Med. 41, e191–e199. 10.1097/CCM.0b013e31828a25d6 23887232

[B108] Sebaa S. Boucherit-Otmani Z. Courtois P. (2019). Effects of tyrosol and farnesol on Candida albicans biofilm. Mol. Med. Rep. 19, 3201–3209. 10.3892/mmr.2019.9981 30816484 PMC6423612

[B109] Seman B. G. Moore J. L. Scherer A. K. Blair B. A. Manandhar S. Jones J. M. (2018). Yeast and filaments have specialized, independent activities in a zebrafish model of Candida albicans infection. Infect. Immun. 86, e00415–18. 10.1128/IAI.00415-18 PMC6204735 30037799

[B110] Shannon P. Markiel A. Ozier O. Baliga N. S. Wang J. T. Ramage D. (2003). Cytoscape: a software environment for integrated models of biomolecular interaction networks. Genome Res. 13, 2498–2504. 10.1101/gr.1239303 14597658 PMC403769

[B111] Shirtliff M. E. Peters B. M. Jabra-Rizk M. A. (2009). Cross-kingdom interactions: Candida albicans and bacteria. FEMS Microbiol. Lett. 299, 1–8. 10.1111/j.1574-6968.2009.01668.x 19552706 PMC4406406

[B112] Short F. L. Murdoch S. L. Ryan R. P. (2014). Polybacterial human disease: The ills of social networking. Trends Microbiol. 22, 508–516. 10.1016/j.tim.2014.05.007 24938173 PMC4158425

[B113] Shrout J. D. Chopp D. L. Just C. L. Hentzer M. Givskov M. Parsek M. R. (2006). The impact of quorum sensing and swarming motility on *Pseudomonas aeruginosa* biofilm formation is nutritionally conditional. Mol. Microbiol. 62, 1264–1277. 10.1111/j.1365-2958.2006.05421.x 17059568

[B114] Soheili V. Tajani A. S. Ghodsi R. Bazzaz B. S. F. (2019). Anti-PqsR compounds as next-generation antibacterial agents against Pseudomonas aeruginosa: A review. Eur. J. Med. Chem. 172, 26–35. 10.1016/j.ejmech.2019.03.049 30939351

[B115] Strateva T. Mitov I. (2011). Contribution of an arsenal of virulence factors to pathogenesis of Pseudomonas aeruginosa infections. Ann. Microbiol. 61, 717–732. 10.1007/s13213-011-0273-y

[B116] Sudbery P. E. (2011). Growth of Candida albicans hyphae. Nat. Rev. Microbiol. 9, 737–748. 10.1038/nrmicro2636 21844880

[B117] Totsika M. (2016). Benefits and challenges of antivirulence antimicrobials at the dawn of the post-antibiotic era. Drug Deliv. Lett. 6, 30–37. 10.2174/22103031066661605061200

[B118] Trejo-Hernández A. Andrade-Domínguez A. Hernández M. Encarnación S. (2014). Interspecies competition triggers virulence and mutability in Candida albicans-Pseudomonas aeruginosa mixed biofilms. ISME J. 8, 1974–1988. 10.1038/ismej.2014.53 24739628 PMC4184018

[B119] Tupe S. G. Kulkarni R. R. Shirazi F. Sant D. G. Joshi S. P. Deshpande M. V. (2015). Possible mechanism of antifungal phenazine-1-carboxamide from Pseudomonas sp. against dimorphic fungi Benjaminiella poitrasii and human pathogen Candida albicans. J. Appl. Microbiol. 118, 39–48. 10.1111/jam.12675 25348290

[B120] Ueda A. Wood T. K. (2009). Connecting quorum sensing, c-di-GMP, pel polysaccharide, and biofilm formation in *Pseudomonas aeruginosa* through tyrosine phosphatase TpbA (PA3885). PloS Pathog. 5, e1000483. 10.1371/journal.ppat.1000483 19543378 PMC2691606

[B121] Vylkova S. Lorenz M. C. (2017). Phagosomal neutralization by the fungal pathogen Candida albicans induces macrophage pyroptosis. Infect. Immun. 85, e00832–16. 10.1128/IAI.00832-16 PMC5278172 27872238

[B122] Wall G. Montelongo-Jauregui D. Vidal Bonifacio B. Lopez-Ribot J. L. Uppuluri P. (2019). Candida albicans biofilm growth and dispersal: contributions to pathogenesis. Curr. Opin. Microbiol. 52, 1–6. 10.1016/j.mib.2019.04.001 31085405 PMC6842673

[B123] Watrous J. D. Phelan V. V. Hsu C. C. Moree W. J. Duggan B. M. Alexandrov T. (2013). Microbial metabolic exchange in 3D. ISME J. 7, 770–780. 10.1038/ismej.2012.155 23283018 PMC3603389

[B124] Westwater C. Balish E. Schofield D. A. (2005). Candida albicans-Conditioned Medium Protects Yeast Cells from Oxidative Stress: a Possible Link between Quorum Sensing and Oxidative Stress Resistance. Eukaryot. Cell 4, 1654–1661. 10.1128/EC.4.10.1654-1661.2005 16215173 PMC1265892

[B125] WHO (2017). Guidelines for the prevention and control of carbapenem-resistant Enterobacteriaceae, Acinetobacter baumannii and Pseudomonas aeruginosa in health care facilities. World Health Organization Available at: https://www.who.int/infection-prevention/publications/guidelines-cre/en/. 29630191

[B126] Wongsuk T. Pumeesat P. Luplertlop N. (2016). Fungal quorum sensing molecules: Role in fungal morphogenesis and pathogenicity. J. Basic Microbiol. 56, 440–447. 10.1002/jobm.201500759 26972663

