# Supplementary material for: Unraveling Pseudomonas aeruginosa and Candida albicans Communication in Coinfection Scenarios: Insights Through Network Analysis
Source: Front Cell Infect Microbiol. 2020 Nov 11;10:550505. doi: 10.3389/fcimb.2020.550505 (PMC7686562; doi:10.3389/fcimb.2020.550505)
Supplement: Supplementary file 1 [file DataSheet_1.docx]

**Reference List of the 29 Relevant Papers Annotated**

Abdel-Rhman, S. H., El-Mahdy, A. M., and El-Mowafy, M. (2015). Effect of Tyrosol and Farnesol on Virulence and Antibiotic Resistance of Clinical Isolates of Pseudomonas aeruginosa. Biomed Res. Int. 2015, 456463. doi:10.1155/2015/456463.

Bandara, H. M. H. N., K Cheung, B. P., Watt, R. M., Jin, L. J., and Samaranayake, L. P. (2013). Pseudomonas aeruginosa lipopolysaccharide inhibits Candida albicans hyphae formation and alters gene expression during biofilm development. Mol. Oral Microbiol. 28, 54–69. doi:10.1111/omi.12006.

Bandara, H. M. H. N., Wood, D. L. A., Vanwonterghem, I., Hugenholtz, P., Cheung, B. P. K., and Samaranayake, L. P. (2020). Fluconazole resistance in Candida albicans is induced by Pseudomonas aeruginosa quorum sensing. Sci. Rep. 10. doi:10.1038/s41598-020-64761-3.

Bandara, H. M., Yau, J. Y., Watt, R. M., Jin, L. J., and Samaranayake, L. P. (2010). Pseudomonas aeruginosa inhibits in-vitro Candida biofilm development. BMC Microbiol. 10, 125. doi:10.1186/1471-2180-10-125.

Bergeron, A. C., Seman, B. G., Hammond, J. H., Archambault, L. S., Hogan, D. A., and Wheeler, R. T. (2017). Candida albicans and Pseudomonas aeruginosa interact to enhance virulence of mucosal infection in transparent zebrafish. Infect. Immun. 85. doi:10.1128/IAI.00475-17.

Brand, A., Barnes, J. D., Mackenzie, K. S., Odds, F. C., and Gow, N. A. R. (2008). Cell wall glycans and soluble factors determine the interactions between the hyphae of Candida albicans and Pseudomonas aeruginosa. FEMS Microbiol. Lett. 287, 48–55. doi:10.1111/j.1574-6968.2008.01301.x.

Chen, A. I., Dolben, E. F., Okegbe, C., Harty, C. E., Golub, Y., Thao, S., et al. (2014). Candida albicans Ethanol Stimulates Pseudomonas aeruginosa WspR-Controlled Biofilm Formation as Part of a Cyclic Relationship Involving Phenazines. PLoS Pathog. 10. doi:10.1371/journal.ppat.1004480.

Cugini, C., Calfee, M. W., Farrow, J. M., Morales, D. K., Pesci, E. C., and Hogan, D. A. (2007). Farnesol, a common sesquiterpene, inhibits PQS production in Pseudomonas aeruginosa. Mol. Microbiol. 65, 896–906. doi:10.1111/j.1365-2958.2007.05840.x.

Cugini, C., Morales, D. K., and Hogan, D. A. (2010). Candida albicans-produced farnesol stimulates Pseudomonas quinolone signal production in LasR-defective Pseudomonas aeruginosa strains. Microbiology 156, 3096–3107. doi:10.1099/mic.0.037911-0.

Curutiu, C., Ditu, L. M., Iordache, F., Bleotu, C., Chifiriuc, M. C., Lazar, V., et al. (2017). Quorum Sensing molecules produced by Pseudomonas aeruginosa impair attachment and biofilm formation in Candida albicans. Biointerface Res. Appl. Chem. 7, 2016–2020. Available at: http://biointerfaceresearch.com/?download=2000%0A.

Davies, D. G., and Marques, C. N. H. (2009). A fatty acid messenger is responsible for inducing dispersion in microbial biofilms. J. Bacteriol. 191, 1393–1403. doi:10.1128/JB.01214-08.

Fourie, R., Ells, R., Kemp, G., Sebolai, O. M., Albertyn, J., and Pohl, C. H. (2017). Pseudomonas aeruginosa produces aspirin insensitive eicosanoids and contributes to the eicosanoid profile of polymicrobial biofilms with Candida albicans. Prostaglandins Leukot. Essent. Fat. Acids 117, 36–46. doi:10.1016/j.plefa.2017.01.008.

Fox, S. J., Shelton, B. T., and Kruppa, M. D. (2013). Characterization of Genetic Determinants That Modulate Candida albicans Filamentation in the Presence of Bacteria. PLoS One 8. doi:10.1371/journal.pone.0071939.

Hall, R. A., Turner, K. J., Chaloupka, J., Cottier, F., de Sordi, L., Sanglard, D., et al. (2011). The quorum-sensing molecules farnesol/homoserine lactone and dodecanol operate via distinct modes of action in candida albicans. Eukaryot. Cell 10, 1034–1042. doi:10.1128/EC.05060-11.

Hogan, D. A., Vik, Å., and Kolter, R. (2004). A Pseudomonas aeruginosa quorum-sensing molecule influences Candida albicans morphology. Mol. Microbiol. 54, 1212–1223. doi:10.1111/j.1365-2958.2004.04349.x.

Holcombe, L. J., McAlester, G., Munro, C. A., Enjalbert, B., Brown, A. J. P., Gow, N. A. R., et al. (2010). Pseudomonas aeruginosa secreted factors impair biofilm development in Candida albicans. Microbiology 156, 1476–1485. doi:10.1099/mic.0.037549-0.

Lopez-Medina, E., Fan, D., Coughlin, L. A., Ho, E. X., Lamont, I. L., Reimmann, C., et al. (2015). Candida albicans Inhibits Pseudomonas aeruginosa Virulence through Suppression of Pyochelin and Pyoverdine Biosynthesis. PLoS Pathog. 11. doi:10.1371/journal.ppat.1005129.

McAlester, G., O’Gara, F., and Morrissey, J. P. (2008). Signal-mediated interactions between Pseudomonas aeruginosa and Candida albicans. J. Med. Microbiol. 57, 563–569. doi:10.1099/jmm.0.47705-0.

Morales, D. K., Jacobs, N. J., Rajamani, S., Krishnamurthy, M., Cubillos-Ruiz, J. R., and Hogan, D. A. (2010). Antifungal mechanisms by which a novel Pseudomonas aeruginosa phenazine toxin kills Candida albicans in biofilms. Mol. Microbiol. 78, 1379–1392. doi:10.1111/j.1365-2958.2010.07414.x.

O’Brien, T. J., and Welch, M. (2019). A Continuous-Flow Model for in vitro Cultivation of Mixed Microbial Populations Associated With Cystic Fibrosis Airway Infections. Front. Microbiol. 10. doi:10.3389/fmicb.2019.02713.

Park, S. J., Han, K. H., Park, J. Y., Choi, S. J., and Lee, K. H. (2014). Influence of bacterial presence on biofilm formation of Candida albicans. Yonsei Med. J. 55, 449–458. doi:10.3349/ymj.2014.55.2.449.

Pires, D. P., Silva, S., Almeida, C., Henriques, M., Anderson, E. M., Lam, J. S., et al. (2013). Evaluation of the ability of C. albicans to form biofilm in the presence of phage-resistant phenotypes of P. aeruginosa. Biofouling 29, 1169–1180. doi:10.1080/08927014.2013.831842.

Purschke, F. G., Hiller, E., Trick, I., and Rupp, S. (2012). Flexible survival strategies of Pseudomonas aeruginosa in biofilms result in increased fitness compared with Candida albicans. Mol. Cell. Proteomics 11, 1652–1669. doi:10.1074/mcp.M112.017673.

Reen, F. J., Mooij, M. J., Holcombe, L. J., Mcsweeney, C. M., Mcglacken, G. P., Morrissey, J. P., et al. (2011). The Pseudomonas quinolone signal (PQS), and its precursor HHQ, modulate interspecies and interkingdom behaviour. FEMS Microbiol. Ecol. 77, 413–428. doi:10.1111/j.1574-6941.2011.01121.x.

Thein, Z. M., Samaranayake, Y. H., and Samaranayake, L. P. (2006). Effect of oral bacteria on growth and survival of Candida albicans biofilms. Arch. Oral Biol. 51, 672–680. doi:10.1016/j.archoralbio.2006.02.005.

Trejo-Hernández, A., Andrade-Domínguez, A., Hernández, M., and Encarnación, S. (2014). Interspecies competition triggers virulence and mutability in Candida albicans-Pseudomonas aeruginosa mixed biofilms. ISME J. 8, 1974–1988. doi:10.1038/ismej.2014.53.

Watrous, J. D., Phelan, V. V., Hsu, C. C., Moree, W. J., Duggan, B. M., Alexandrov, T., et al. (2013). Microbial metabolic exchange in 3D. ISME J. 7, 770–780. doi:10.1038/ismej.2012.155.

Xu, L. Q., Zeng, J. W., Jiang, C. H., Wang, H., Li, Y. Z., Wen, W. H., et al. (2017). Isolation and determination of four potential antimicrobial components from Pseudomonas aeruginosa extracts. Int. J. Med. Sci. 14, 1368–1374. doi:10.7150/ijms.18896.

Xu, L., Wang, F., Shen, Y., Hou, H., Liu, W., Liu, C., et al. (2014). Pseudomonas aeruginosa inhibits the growth of pathogenic fungi: In vitro and in vivo studies. Exp. Ther. Med. 7, 1516–1520. doi:10.3892/etm.2014.1631.
